# Interleukin-21 receptor signaling promotes metabolic dysfunction-associated steatohepatitis-driven hepatocellular carcinoma by inducing immunosuppressive IgA^+^ B cells

**DOI:** 10.1186/s12943-024-02001-2

**Published:** 2024-05-08

**Authors:** Ying Xie, Yu Huang, Zhi-Yong Li, Weihua Jiang, Nan-Xi Shi, Yuanzhi Lu, Guangchao Cao, Zhinan Yin, Xue-Jia Lin

**Affiliations:** 1 The Biomedical Translational Research Institute, Key Laboratory of Viral Pathogenesis & Infection Prevention and Control, School of Medicine, Jinan University, Guangzhou, 510632 China; 2 Guangdong Provincial Key Laboratory of Tumor Interventional Diagnosis and Treatment, Zhuhai Institute of Translational Medicine, Zhuhai People’s Hospital Affiliated With Jinan University, Jinan University, Zhuhai, 519000 China; 3 Department of Pathology, The First Affiliated Hospital of Jinan University, Jinan University, Guangzhou, 510632 China

**Keywords:** IL-21R, MASH-driven HCC, IgA^+^ B cell, Cancer-promoting role

## Abstract

**Background:**

Dysregulation of immune surveillance is tightly linked to the development of metabolic dysfunction-associated steatohepatitis (MASH)-driven hepatocellular carcinoma (HCC); however, its underlying mechanisms remain unclear. Herein, we aimed to determine the role of interleukin-21 receptor (IL-21R) in MASH-driven HCC.

**Methods:**

The clinical significance of IL-21R was assessed in human HCC specimens using immunohistochemistry staining. Furthermore, the expression of IL-21R in mice was assessed in the STAM model. Thereafter, two different MASH-driven HCC mouse models were applied between IL-21R-deficient mice and wild type controls to explore the role of IL-21R in MASH-driven HCC. To further elucidate the potential mechanisms by which IL-21R affected MASH-driven HCC, whole transcriptome sequencing, flow cytometry and adoptive lymphocyte transfer were performed. Finally, flow cytometry, enzyme-linked immunosorbent assay, immunofluorescent staining, chromatin immunoprecipitation assay and western blotting were conducted to explore the mechanism by which IL-21R induced IgA^+^ B cells.

**Results:**

HCC patients with high IL-21R expression exhibited poor relapse-free survival, advanced TNM stage and severe steatosis. Additionally, IL-21R was demonstrated to be upregulated in mouse liver tumors. Particularly, ablation of IL-21R impeded MASH-driven hepatocarcinogenesis with dramatically reduction of lipid accumulation. Moreover, cytotoxic CD8^+^ T lymphocyte activation was enhanced in the absence of IL-21R due to the reduction of immunosuppressive IgA^+^ B cells. Mechanistically, the IL-21R-STAT1-c-Jun/c-Fos regulatory axis was activated in MASH-driven HCC and thus promoted the transcription of *Igha*, resulting in the induction of IgA^+^ B cells.

**Conclusions:**

IL-21R plays a cancer-promoting role by inducing IgA^+^ B cells in MASH-driven hepatocarcinogenesis. Targeting IL-21R signaling represents a potential therapeutic strategy for cancer therapy.

**Supplementary Information:**

The online version contains supplementary material available at 10.1186/s12943-024-02001-2.

## Background

Hepatocellular carcinoma (HCC) is the most common malignancy, with the fifth highest incidence and third highest mortality among malignant tumors around the world. Viral infections, alcohol consumption and obesity are the main causes of HCC. With recent advances in the prevention and treatment of hepatitis B virus (HBV) and hepatitis C virus (HCV) infection, the burden of HCC due to viral hepatitis is declining; however, the prevalence of HCC caused by metabolic dysfunction-associated steatotic liver disease (MASLD) [1], previously known as non-alcoholic fatty liver disease (NAFLD), is clearly increasing [2, 3]. Globally, a quarter of the population has NAFLD [2], and approximately 20% of these individuals harbor non-alcoholic steatohepatitis (NASH), now referred to as metabolic dysfunction-associated steatohepatitis (MASH) [1]. MASH, whose key features are steatosis, fibrosis and chronic inflammation [4, 5], can progress to cirrhosis and further to HCC [3]. Generally, MASH is the liver manifestation of metabolic syndrome and is associated with obesity, insulin resistance and dyslipidemia [2, 6], although lean MASH patients that doesn’t fit this definition can be also diagnosed. Although the percentage of patients diagnosed annually with MASH-associated HCC is still relatively low, MASH will inevitably become the most common etiology of HCC in the near future due to the rapidly increasing incidence of obesity and diabetes globally [7]. Thus, more extensive exploration on the tumorigenesis of MASH-associated HCC is urgently needed to develop efficient therapeutic strategies to prevent the progression from MASH to HCC.

Emerging investigations imply that MASH-driven HCC development is accompanied by the accumulation of immune cells within the tumor microenvironment, playing an important role in initiating, maintaining, or exacerbating the transition from MASH to HCC [8]. Dysregulation of immune surveillance is proposed to be one of the new mechanisms that have been implicated in MASH-driven HCC [2]. For instance, Kupffer cells, the liver resident macrophages, represent a first-line defense force in the liver; however, they have been shown to lack effective turnover in MASH [9] and are likely to turn into tumor-associated macrophages (TAMs) to fuel a tumor-promoting inflammatory response under the fibrotic and steatotic tumor environment [10]. In addition, other innate immune cells, such as dendritic cells (DCs) [11] and natural killer (NK) cells [12] may also participate in the process of MASH-driven HCC. Notably, adaptive CD8^+^ T cells play a central role in the hepatocarcinogenesis in the context of MASLD. Recently, IL-15 produced in the hepatic microenvironment is revealed to downregulate FOXO1 in CD8^+^ T cells, enabling them to acquire a resident character by upregulating CXCR6, thus making CXCR6^+^PD1^+^CD8^+^ T cells to be capable of eliciting auto-aggressive killing of liver cells to trigger MASH and the transition to HCC [13]. Furthermore, anti–PD-1 therapy fails to reduce the tumor burden in preclinical models of MASH-related HCC and indeed results in the accumulation of CXCR6^+^PD1^+^CD8^+^ T cells [14], suggesting that these cells are likely responsible for the lack of responsiveness of MASH-related HCC to immune checkpoint inhibitors. Compared to CD8^+^ T cells, naïve CD4^+^ T cells are more vulnerable to the MASLD microenvironment, where they display higher mortality rate due to the oxidative stress–related cytotoxic effects exerted by free fatty acids [15]. Hence, the loss of CD4^+^ T cells leads to failure of cancer surveillance in MASH-induced HCC [15]. However, regulatory T (Treg) cells, a subset of CD4^+^ T cells, are increased in the liver of MASH-induced HCC and promote carcinogenesis by supporting an immunosuppressive microenvironment [16]. Growing evidences clarify the contribution of B cells to MASH as well as MASH-to-HCC transition, and we have also demonstrated that inflammation-induced immunosuppressive IgA^+^ B cells dismantle anti-cancer immunity by suppressing cytotoxic CD8^+^ T lymphocyte (CTL) activation in MASH-driven HCC [17]. However, the mechanism of producing of IgA^+^ B cells is still yet to be explored.

Interleukin-21 (IL-21), a member of the γ chain (γ_c_) cytokine family, is produced mainly by T cells and natural killer T (NKT) cells [18]. Its private receptor, IL-21 receptor (IL-21R), which activates Janus kinase (JAK)-signal transducers and activators of transcription (STAT) signaling upon ligand binding, is expressed by multiple immune cell subsets including, but not limited to, B cells, T cells, NK cells, macrophages and DCs [18, 19]. IL-21/IL-21R signaling plays critical role in immune responses and has been implicated in the regulation of inflammation in various acute and chronic inflammatory diseases, such as cancer [20]. However, the role of IL-21/IL-21R in cancer development remains controversial and has not been extensively investigated in faithful in vivo models. IL-21 was originally demonstrated to be a growth and survival factor in human myeloma cell lines, which is mediated through the activation of the JAK1/STAT3 signaling [21]. In addition, IL-21 has been demonstrated to be immunosuppressive because of its ability to induce IL-10. However, a large number of reports have shown that IL-21 promotes tumor clearance, rather than tumor survival, suggesting that IL-21 is a promising immunotherapeutic agent for cancer treatment [18, 19]. Although limited studies have showed that IL-21/IL-21R is involved in the development of HBV-related diseases, including HCC [22–26]. All of these studies are in vitro or xenograft mouse models studies, lacking of models for spontaneous tumorigenesis in vivo, which is important for studying mechanisms relying on a complicated tumor microenvironment and chronic inflammation. Thus, more extensive exploration for the function of IL-21/IL-21R in HCC tumorigenesis is required. Importantly, IL-21/IL-21R plays a pivotal role in the production of IgA^+^ B cells [27–31], which are identified as immunosuppressive cells in MASH-driven HCC [17], indicating that IL-21/IL-21R signaling may be involved in the tumorigenesis of MASH-driven HCC. However, the function and underlying mechanism of IL-21/IL-21R in MASH-driven hepatocarcinogenesis are completely unknown.

Herein, we clarified the role of IL-21R in MASH-driven HCC and the underlying mechanism of producing IgA^+^ B cells. The results revealed that IL-21R played a cancer-promoting role by activating the IL-21R-STAT1-c-Jun/c-Fos regulatory axis, resulting in the production of immunosuppressive IgA^+^ B cells, and thus attenuated CTL activation in the tumorigenesis of MASH-driven HCC.

## Methods

### Human tissues

Human tumor tissue sections were obtained from 69 histologically confirmed HCC patients at The First Affiliated Hospital of Jinan University (JNU). The relevant characteristics of the 69 participants are summarized in Table S1. The study was approved by the Institutional Ethics Committee at The First Affiliated Hospital of JNU. Informed consent was obtained from all the participants.

### Mouse studies

C57BL/6 control mice and IL-21R-deficient (*Il21r*^*−/−*^) mice were purchased from Biocytogen Pharmaceuticals (Beijing, China) and Jackson Laboratory (JAX), respectively. The genotypes of the *Il21r*^*−/−*^ mice were identified according to the genotyping protocol on the website of JAX (https://www.jax.org/Protocol?stockNumber=019115&protocolID=25258). All mice were bred and maintained in filter-topped cages on autoclaved food and water at the JNU animal facility. All procedures for animal experiments were performed in accordance with the Guide for the Care and Use of Laboratory Animals (NIH publications Nos. 80–23, revised 1996) and according to the Animal Ethics Committee of JNU.

For the STAM model, *Il21r*^*−/−*^ mice and wild type control mice were performed as previously described [32]. Briefly, male mouse pups were subcutaneously injected with 200 μg of streptozocin (STZ, #S0130, Sigma-Aldrich, St. Louis, MO, USA) at two days after birth and fed with high-fat diet (HFD) containing 60 kcal% fat (#D12492, Research Diets, New Brunswick, NJ, USA) at four weeks of age. The mice developed to MASH and HCC at eight and 20 weeks of age, respectively.

For the WD&High sugar solution&CCl_4_ model, *Il21r*^*−/−*^ mice and wild type control mice were performed as previously described [33]. Briefly, four-weeks-old male mice were begun to feed with western diet (WD) containing 21.2% fat, 41% sucrose, and 1.25% cholesterol (#TD. 120528, Harlan, Madison, WI, USA) and a high sugar solution containing 23.1 g/L d-fructose (#F0127, Sigma-Aldrich, St. Louis, MO, USA) and 18.9 g/L d-glucose (#G8270, Sigma-Aldrich, St. Louis, MO, USA). Simultaneously, CCl_4_ dissolved in corn oil was injected intraperitoneally at the dose of 0.2 μL/g of body weight once per week. The mice developed to MASH and HCC at 12 and 24 weeks of age, respectively.

For adoptive lymphocyte transfer, male mice with HCC tumors were intravenously injected via the inner canthus veniplex with 200 μg anti-mouse CD20 antibody (#BE0356, Clone MB20-11, Bioxcell, Lebanon, NH, USA) per mouse to deplete B cells in vivo. Two days later, single-cell suspensions of spleens from either *Il21r*^*−/−*^ mice or wild type control mice were incubated with biotin-labeled anti-mouse CD19 antibody (#115504, Clone 6D5, Biolegend, San Diego, CA, USA), and the CD19^+^ B cells were isolated from the suspensions by using Streptavidin Particles Plus-DM (#557812, BD Biosciences, San Diego, CA, USA). The isolated CD19^+^ B cells (5 × 10^6^ cells per mouse) were randomly transferred into the mice previously treated with anti-mouse CD20 antibody by inner canthus veniplex injection. Mice were sacrificed seven weeks after lymphocyte transfer.

For antibody neutralization experiment, male mice with HCC tumors were injected intraperitoneally with 200 μg anti-mouse IL-21R antibody (#BE0258, Clone 4A9, Bioxcell, Lebanon, NH, USA) or its isotype control (#A2123, Clone 2A3, Selleckchem, Houston, TX, USA) twice a week for five weeks.

### Analysis of gene expression

The expression levels of target genes were assessed by western blotting, immunohistochemistry (IHC) staining, immunofluorescent (IF) staining or real time quantitative RT-PCR (qRT-PCR). The antibodies used for western blotting, IHC and IF staining are listed in Table S2, and the primers used for qRT-PCR are listed in Table S3.

### Chromatin immunoprecipitation (ChIP) assay

CD19^+^ B cells were isolated from splenocytes by using biotin-labeled anti-mouse CD19 antibody and Streptavidin Particles Plus-DM. The isolated CD19^+^ B cells (~ 3 × 10^7^) were subsequently cross-linked with formaldehyde, and the reaction was stopped with glycine. The cross-linked cells were washed with cold 1 × phosphate-buffered saline (PBS) and the pellets were re-suspended with 300 μL of ChIP cell lysis buffer supplemented with protease inhibitor [34], and incubated on ice for 10 min. The lysed cells were centrifuged and the pellets were re-suspended with 300 μL of MNase digestion buffer [34], followed by digestion with MNase (#10011, Cell Signaling Technology, Danvers, MA, USA) (100 gel units/1 × 10^7^ cells) at 37 °C for exactly 10 min, mixing by inversion every 2.5 min. After stopping digestion, the suspensions were centrifuged and the pellets were with re-suspended with 400 μL of ChIP dilution buffer [34] supplemented with protease inhibitor, followed by sonication (amplitude 2, processed time 30 s, pulse ON 5 s, pulse OFF 30 s). Thereafter, the chromatin complexes were immunoprecipitated using anti-c-Jun (#9165S, Cell Signaling Technology, Danvers, MA, USA), anti-c-Fos (#4384S, Cell Signaling Technology, Danvers, MA, USA) or the corresponding isotype-matched IgG overnight and then collected by incubation with Protein A/G Magnetic Beads (#88803, Thermo Fisher Pierce, Waltham, MA, USA) for two hours. The DNA–protein-beads complex was washed and then eluted. Subsequently, the DNA–protein cross-link was reversed by heating, and DNA was purified from the eluted solution and subjected to qRT-PCR using primers covering the activating protein 1 (AP-1) binding sites. The sequences of the primers are listed in Table S3.

### Whole transcriptome sequencing processing and analysis

Total RNA was extracted from the livers or tumors using TRI reagent (#T9424, Sigma-Aldrich, St. Louis, MO, USA), and was subsequently sent to BGI Tech Solutions Co., Ltd (Shenzhen, China) for whole transcriptome sequencing according to their standardized procedures.

For differential expression analysis, the sequencing data were performed using DESeq2, Q value ≤ 0.05 was used to judge the significance of expression difference. For Gene Ontology (GO) enrichment analysis and Kyoto Encyclopedia of Genes and Genomes (KEGG) enrichment analysis, target genes were subjected to enrichment analysis using phyper, a function of R. The *P*-value was corrected using the Bonferroni method, and a corrected *P*-value ≤ 0.05 was taken as the threshold to define the significantly enriched GO or KEGG terms. Additionally, gene set enrichment analysis (GSEA) [35, 36] was performed to identify the hallmark gene sets according to the instruction. Heirarchical clustering was performed using Cluster 3.0 (Michiel de Hoon, Tokyo, Japan) and the data were visualized using Java TreeView (Eisen Lab, Berkeley, CA, USA).

### Statistical analysis

Data were presented as mean ± SEM (standard error of the mean). The differences between two groups were analyzed by Student's *t* test. One-way analysis of variance (ANOVA) was used to compare three or more groups, while two-way ANOVA was used to compare the differences between groups that have been split on two independent categorical variables. All the statistical analyses were performed with GraphPad Prism 6.0 (GraphPad Software, Inc., San Diego, CA, USA). All the statistical tests were two-sided, and *P* < 0.05 was considered statistically significant.

## Results

### IL-21R is increased in MASH-driven HCC and associated with poor prognosis in human HCC patients

**Fig. 1:**
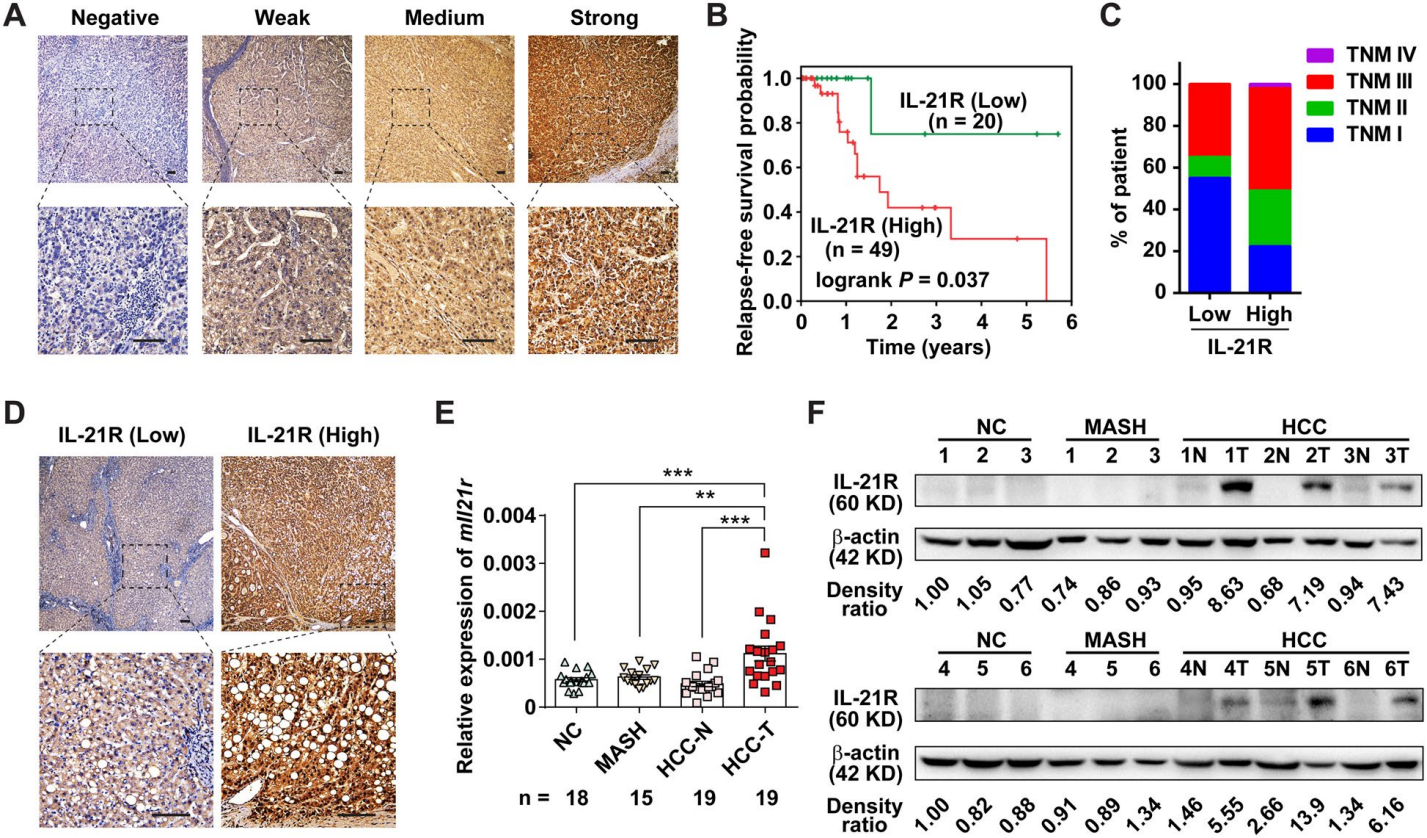
The expression of IL-21R is increased in MASH-driven HCC tissues and higher expression of IL-21R is associated with shorter relapse-free survival in HCC patients. **A** Shown are the representative pictures for immunohistochemistry (IHC) staining with different levels of IL-21R-staining in human HCC tissues. **B** Higher expression of IL-21R was associated with shorter relapse-free survival in HCC patients. The HCC patients (*n* = 69) were divided into IL-21R-low and IL-21R-high groups according to the evaluation scores of IHC in human HCC tissues. **C** The distribution of clinical stages upon diagnosis between HCC patients with low- or high-IL-21R expression. **D** HCC patients with higher expression of IL-21R exhibited more severe steatosis. **E**, **F** The mRNA (**E**) and protein (**F**) levels of IL-21R were increased in mouse MASH-driven HCC. The mRNA or protein levels of IL-21R were detected in mouse liver tissue, paracancerous tissue (N), and cancer tissue (T) from mice at normal chow (NC), metabolic dysfunction-associated steatohepatitis (MASH) and hepatocellular carcinoma (HCC) stages by using qRT-PCR or western blotting. β-actin, internal control. Scale bar = 100μm. The number of patients or mice in each group is shown in B and E accordingly. One-way ANOVA was used to determine significance in E. ** *P* < 0.01, *** *P* < 0.001

While the role of IL-21R signaling has been investigated in infectious and inflammatory diseases, unequivocal evidence about its possible contribution to tumor development in vivo is still poorly understood. To explore the role of IL-21R in HCC, we first assessed the protein level of IL-21R by using IHC staining (Fig. 1A). We found that patients with high IL-21R expression exhibited poor relapse-free survival, more advanced stage of HCC and more liver injury (Fig. 1B, C and Table S1). We further observed that there were 21 patients with different grades of steatosis according to the IHC results. Interestingly, most of these patients expressed high IL-21R (17/21 = 81%), and most of the patients with high IL-21R expression exhibited moderate to severe steatosis (15/17 = 88%), whereas most of the patients with low IL-21R expression displayed mild steatosis (3/4 = 75%, Fig. 1D and Table S4), suggesting that IL-21R might play an important role in MASH-related HCC. Moreover, we evaluated the impact of IL-21R in human HCC patients on relapse-free survival and overall survival using TCGA data. Consistently, we obtained similar results in two independent cohorts (Fig. S1 and Table S5).

Thereafter, we applied a well-established mouse model of MASH-driven HCC (Fig. S2A-E) to investigate the role of IL-21R in MASH-driven HCC. We found that both the mRNA (Fig. 1E) and protein (Fig. 1F and Fig. S2F) levels of IL-21R were upregulated in the liver tumors.

Taken together, these observations in human and mouse HCC samples suggest a potential cancer-promoting role of IL-21R in MASH-driven HCC.

### IL-21R–deficient mice impede MASH-driven HCC development

**Fig. 2:**
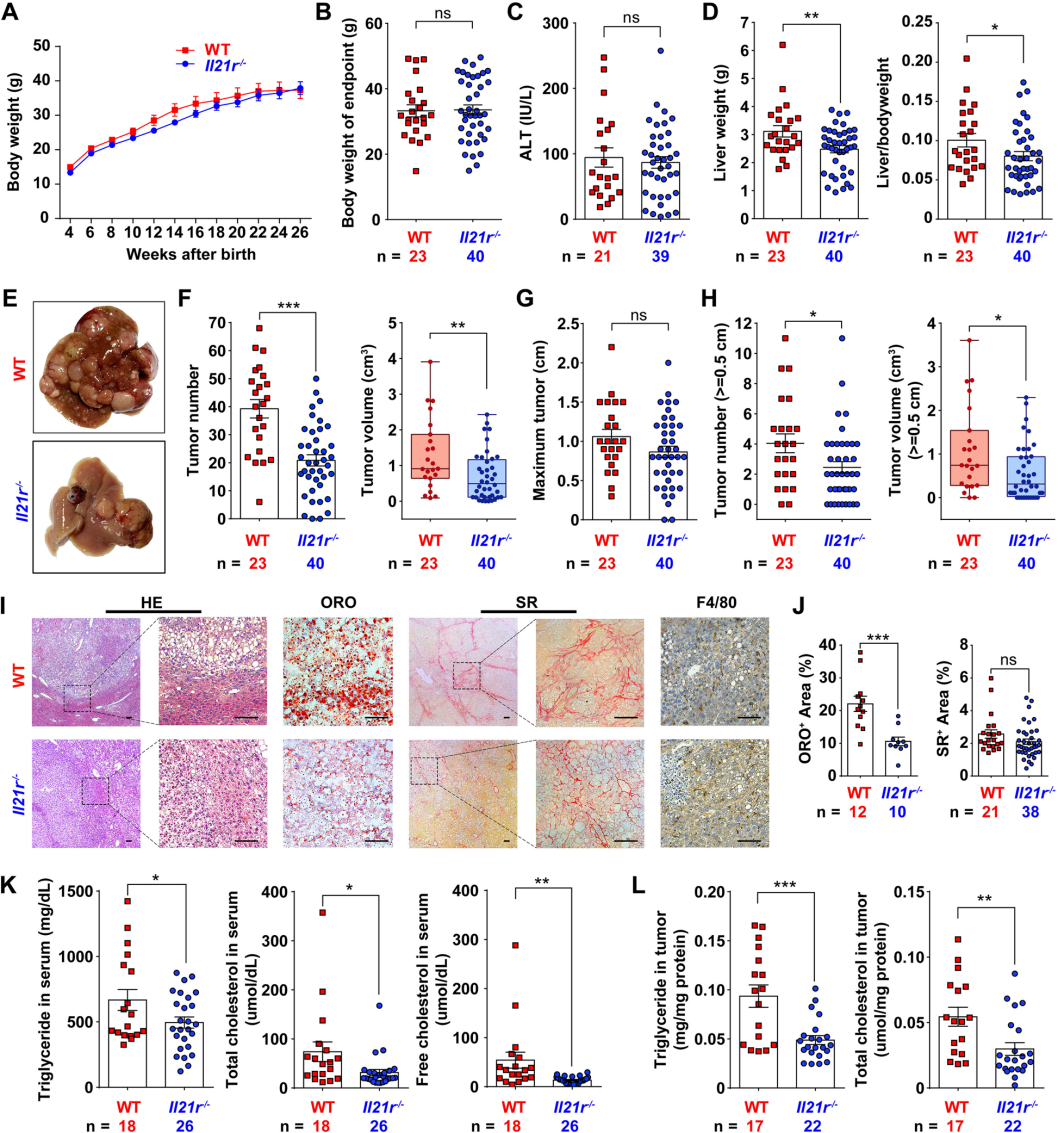
*Il21r*^*−/−*^ mice exhibit less tumor burden of HCC with decreased lipid droplets. **A** The body weight gain of IL-21R*-*deficient (*Il21r*^*−/−*^) mice and wild type (WT) controls in the STAM model. Mice were fed with high-fat diet at four weeks of age, and continuously monitored from four to 27 weeks, and 12 data points were used to analyze weight gain, with 25 mice per time point. **B-H** Comparison of body weight of endpoint (**B**), serum ALT levels (**C**), liver weight, liver/body weight ratio (**D**), representative pictures of liver (**E**), number and volume of tumors (**F**), largest diameter of tumor (**G**), and number and volume of large tumors (**H**) between *Il21r*^*−/−*^ mice and WT controls at 27 weeks of age in the STAM model. **I** Shown are the representative pictures for hematoxylin–eosin (HE), Oil Red O (ORO), Sirius Red (SR) and F4/80 immunohistochemistry staining of the liver/tumor tissues between *Il21r*^*−/−*^ mice and WT controls at 27 weeks of age in the STAM model. Scale bar = 100μm. **J** Lipid droplets were significantly decreased in *Il21r*^*−/−*^ mice. Lipid droplets and collagen deposition were quantified according to the image analysis of ORO and SR staining, respectively. **K**, **L** Triglyceride and cholesterol levels were significantly decreased in the serum or liver tumor extracts of *Il21r*^*−/−*^ mice. Serum (**K**) or liver tumor extracts (**L**) were analyzed for triglyceride, total cholesterol and free cholesterol. The number of mice in each group is shown in panels accordingly. To determine the significance, two-way ANOVA was used in A while Student's *t* test was used in other panels accordingly. * *P* < 0.05, ** *P* < 0.01, *** *P* < 0.001, ns, not significant difference

To determine the role of IL-21R in MASH-driven HCC development, the STAM model was applied between IL-21R-deficient mice (*Il21r*^*−/−*^, Fig. S3A) and wild type (WT) controls. We found that ablation of IL-21R did not affect weight gain, liver damage, colon length and glucose tolerance (Fig. 2A-C and Fig. S3B, C). However, liver weight, HCC tumorigenesis and spleen weight were significantly reduced in *Il21r*^*−/−*^ mice compared to WT controls (Fig. 2D-H and Fig. S3D). Consistent with the limited tumor burden, HCC tumors from IL-21R-deficient mice were also characterized by lower expression of proliferation markers, including cyclin D1 (*Ccnd1*), antigen identified by monoclonal antibody Ki67 (*Mki67*) and cyclin dependent kinase 4 (*Cdk4*) (Fig. S3E), implying reduced proliferative capacity of tumors in the absence of IL-21R, which was further confirmed by IHC staining for Ki67 (Fig. S3F). Nonetheless, ablation of IL-21R did not change the apoptotic cell death (Fig. S3G). We further confirmed that IL-21R-deficient mice impede MASH-driven HCC tumorigenesis in another mouse model of MASH-driven HCC (Fig. S4A-H).

Furthermore, IL-21R-deficient mice showed no obvious alterations in hepatocyte ballooning and Kupffer cells infiltration (Fig. 2I). Importantly, lipid accumulation was dramatically reduced in HCC tumors from *Il21r*^*−/−*^ mice (Fig. 2I, J). Consistently, both triglyceride and cholesterol levels decreased in the serum and tumor tissue of *Il21r*^*−/−*^ mice (Fig. 2K, L). Similar results were obtained using another mouse model of MASH-driven HCC (Fig. S4I-L).

Collectively, these data clearly demonstrate that IL-21R promotes MASH-driven HCC and affects lipid accumulation.

### Ablation of IL-21R enhances cytotoxic CD8^+^ T lymphocyte activation in vivo

**Fig. 3:**
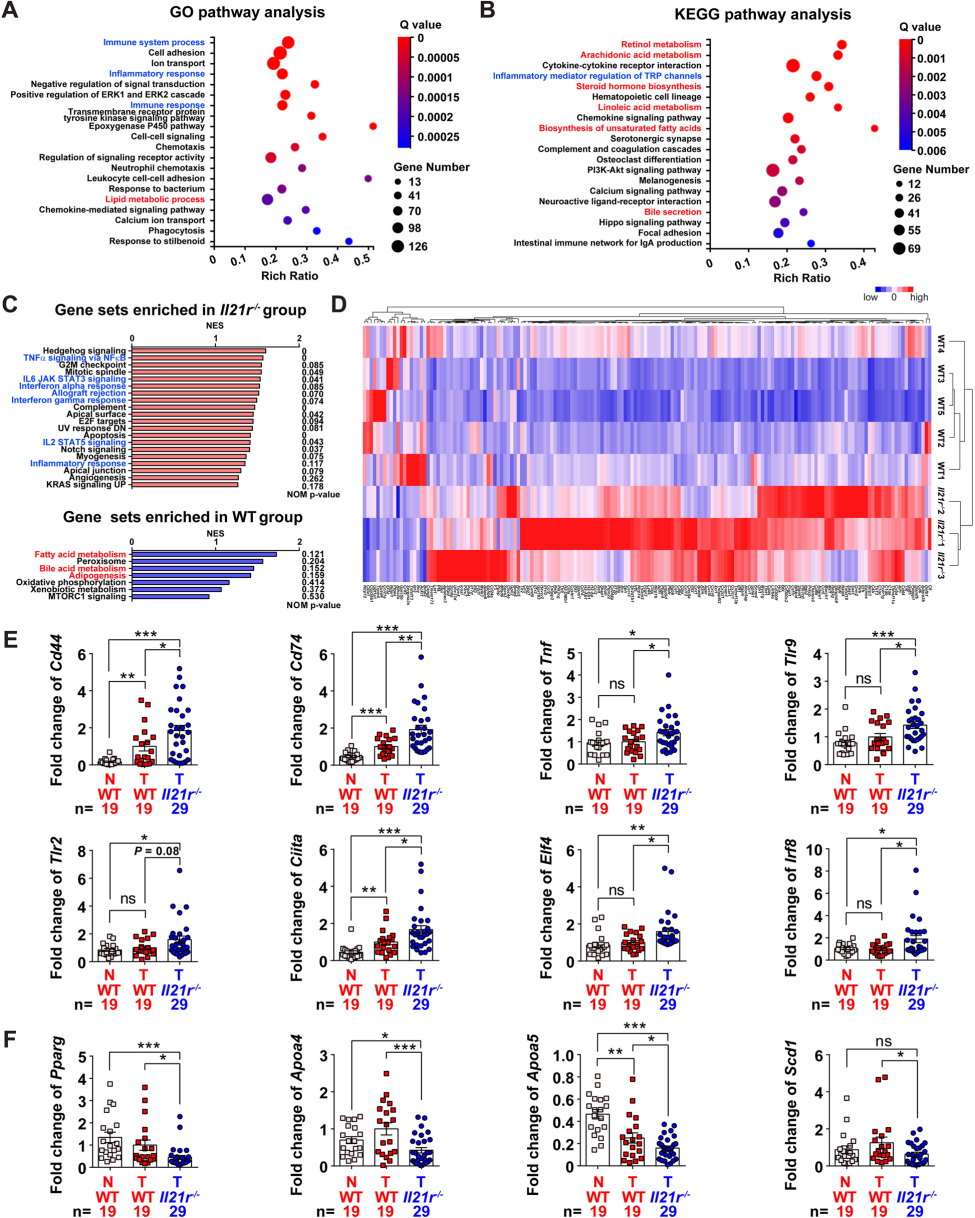
Differential genes between the tumor tissues of *Il21r*^*−/−*^ mice and wild type controls are enriched in immune, inflammatory and metabolic pathways. **A**, **B** GO and KEGG pathway analyses of the differential genes in tumor tissues between *Il21r*^*−/−*^ mice and wild type (WT) controls. The horizontal axis shows the enrichment score. The larger the bubble is, the more differential genes are contained in the entry. The smaller the enrichment Q Value is, the greater the degree of significance. In the vertical axis, the pathways in blue font represent immune- and inflammation-related pathways, whereas the pathways in red font represent metabolism-related pathways. **C** The top hallmark gene sets sorted by normalized enrichment score (NES) are shown to depict gene sets enriched in *Il21r*^*−/−*^ mice (upper panel) and WT controls (lower panel) according to gene set enrichment analysis (GSEA). The immune- and inflammation-related gene sets are colored in blue, whereas the metabolism-related gene sets are colored in red. **D** Heat map depicting the differential expression of 170 immune- and inflammation-related genes in the indicated strains, illustrating the upregulation of most immune- and inflammation-related genes in *Il21r*^*−/−*^ mice. **E**, **F** The mRNA levels of several immune- and inflammation-related genes (**E**) and metabolism-related genes (**F**) were detected in the paracancerous tissue (N), and tumor tissue (T) from *Il21r*^*−/−*^ mice and WT controls. For **E** and **F**, the number of mice in each group is shown. Student's *t* test was used to determine significance. * *P* < 0.05, ** *P* < 0.01, *** *P* < 0.001, ns, not significant difference

To elucidate the potential mechanisms by which IL-21R affects MASH-driven HCC, we first conducted whole transcriptome sequencing on isolated HCC tumors from *Il21r*^*−/−*^ mice and WT controls. GO and KEGG pathway analyses revealed that differential genes were enriched in inflammation-, immune- and metabolism-related pathways (Fig. 3A, B). Further GSEA analysis showed that the *Il21*^*−/−*^ group enriched gene sets related to inflammation and immune, whereas the WT group enriched in metabolism-related gene sets (Fig. 3C and Fig. S5A, B). The inflammation- and immune-related genes with differential expression were clustered (Fig. 3D) and most of them, including *Cd44*, *Cd74*, *Tnf*, were confirmed to be upregulated in the tumors of *Il21r*^*−/−*^ mice (Fig. 3E). We also found that lipid-related genes decreased in the tumors of *Il21r*^*−/−*^ mice, but no alterations of fibrosis-related genes (Fig. 3F and Fig. S5C), confirming the results of histology (Fig. 2I). Moreover, pro-inflammation cytokines, such as *Il1b*, *Il12b* and *Il18*, were upregulated in the tumor of *Il21r*^*−/−*^ mice; whereas anti-inflammation cytokines, including *Il10* and *Il22* were decreased in the tumor of *Il21r*^*−/−*^ mice (Fig. S5D).

**Fig. 4:**
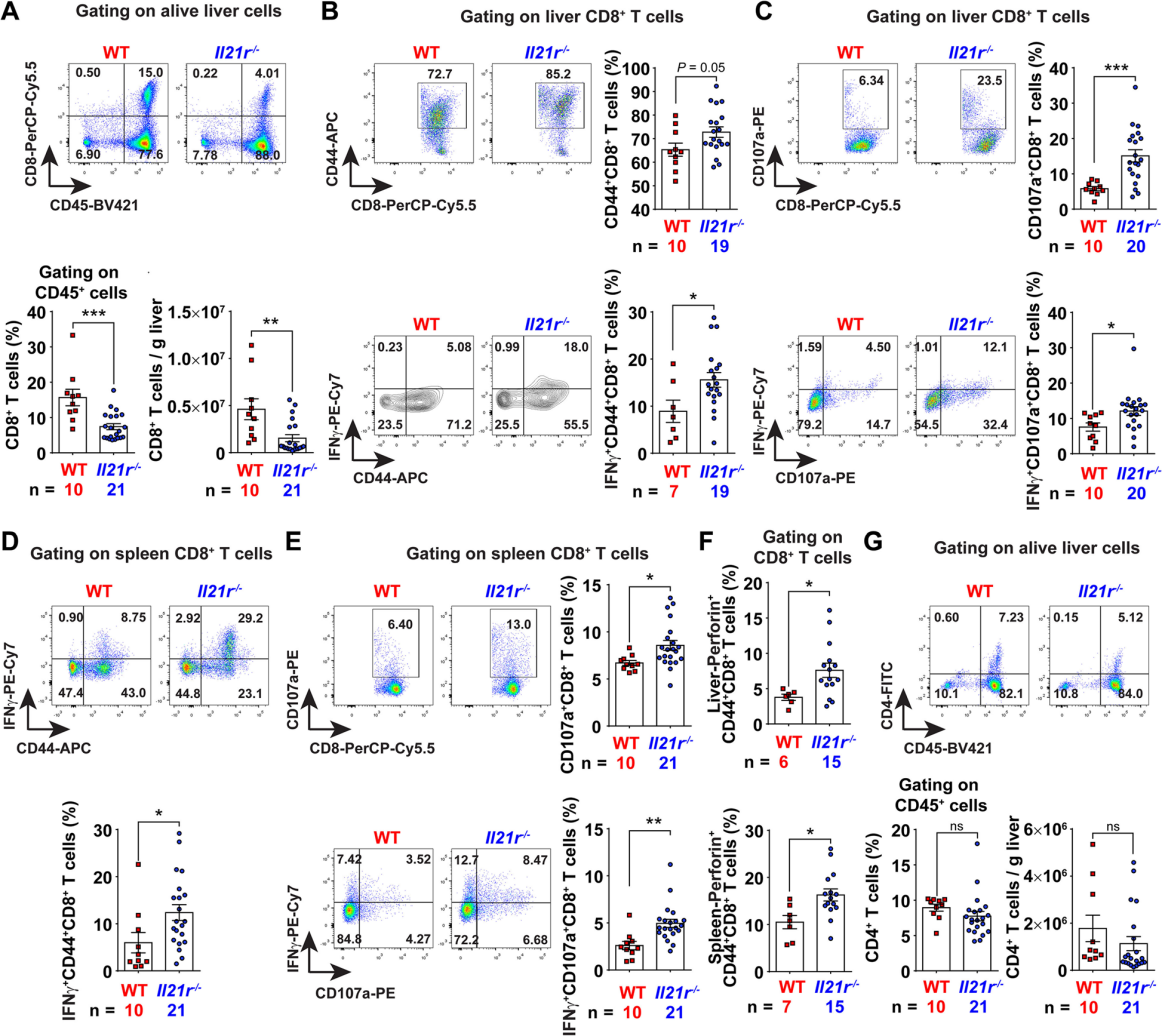
Ablation of IL-21R enhances cytotoxic CD8^+^ T lymphocyte activation in vivo. **A** Liver/tumor CD8^+^ T cells were significantly decreased in *Il21r*^*−/−*^ mice. The representative flow cytometry images are shown in the upper panel. In the lower panel, the percentages of CD8^+^ T cells and the absolute cell number of CD8^+^ T cells per gram of liver/tumor tissues were analyzed between *Il21r*^*−/−*^ mice and wild type (WT) controls. **B** Effector CD8^+^ T cells were significantly increased in the liver/tumor tissues of *Il21r*^*−/−*^ mice. The percentages of CD8^+^ T cells positive for CD44 (upper panel), CD44 and IFNγ (lower panel) in the liver/tumor tissues were analyzed between *Il21r*^*−/−*^ mice and WT controls. **C** Degranulating CD8^+^ T cells were significantly increased in the liver/tumor tissues of *Il21r*^*−/−*^ mice. The percentages of CD8^+^ T cells positive for CD107a (upper panel), CD107a and IFNγ (lower panel) in liver/tumor were analyzed between *Il21r*^*−/−*^ mice and WT controls. **D**, **E** Both effector (**D**) and degranulating (**E**) CD8^+^ T cells were significantly increased in the spleens of *Il21r*^*−/−*^ mice. The percentages of CD8^+^ T cells positive for CD44 and IFNγ (**D**), CD107a, CD107a and IFNγ (**E**) in the spleen were analyzed between *Il21r*^*−/−*^ mice and WT controls. **F** Perforin^+^CD8^+^ T cells were significantly increased in *Il21r*^*−/−*^ mice. **G** Ablation of IL-21R did not change the percentage and number of liver/tumor CD4^+^ T cells. The percentages of CD4^+^ T cells and the absolute cell number of CD4^+^ T cells per gram of liver/tumor tissues were analyzed between *Il21r*^*−/−*^ mice and WT controls. The gating schemes are indicated above each panel, and the number of mice in each group is shown in the panels accordingly. Student's *t* test was used to determine significance. * *P* < 0.05, ** *P* < 0.01, *** *P* < 0.001, ns, not significant difference

In the light of enrichment of inflammation- and immune-related pathways, we next performed flow cytometry (Fig. S6) to explore the difference of immune microenvironment between *Il21r*^*−/−*^ mice and WT controls. Compared with WT controls, *Il21r*^*−/−*^ mice had remarkably fewer liver CD8^+^ T cells (Fig. 4A), but equal numbers of spleen CD8^+^ T cells (Fig. S7A). Nonetheless, compared to WT controls, *Il21r*^*−/−*^ mice had more effector and degranulating CD8^+^ T cells in both liver and spleen (Fig. 4B-F and Fig. S7B), but had a similar percentage of Tim3^+^CD8^+^ exhausted T cells (Fig. S7C). Similar results were obtained using another MASH-driven HCC mouse model (Fig. S7D-F). Additionally, ablation of IL-21R had no effect on total CD4^+^ T cells (Fig. 4G and Fig. S8A), although the numbers of HCC-infiltrating Th1, Th17 and Treg cells increased in the absence of IL-21R (Fig. S8B-E), while having little effect on liver B cells (Fig. S9A).

These data indicate that MASH-driven HCC development is impeded in *Il21r*^*−/−*^ mice mainly by enhanced cytotoxic CTL activation.

### IL-21R-deficient mice have fewer IgA^+^ B cells, and thus impede MASH-driven hepatocarcinogenesis

**Fig. 5:**
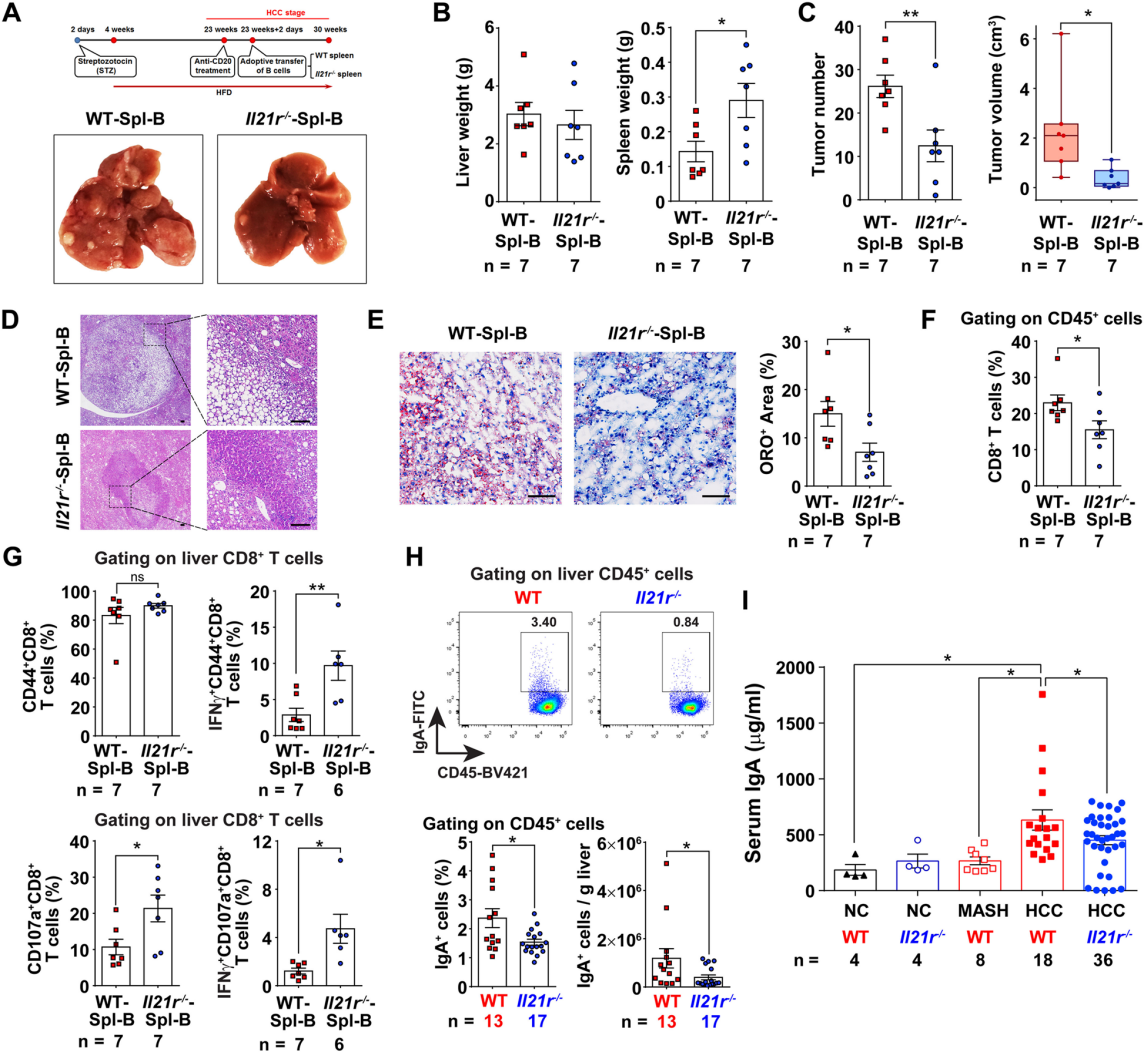
IL-21R-deficient mice have fewer IgA^+^ B cells, and thus impede MASH-driven HCC development. **A** Shown are the schematic diagram of adoptive lymphocyte transfer experiment and the representative pictures of the endpoint liver from mice transferred with B cells from *Il21r*^*−/−*^ mice (*Il21r*^*−/−*^-Spl-B) or WT controls (WT-Spl-B). **B**, **C** Comparison of liver weight, spleen weight (**B**) and the number and volume of tumors (**C**) between the mice transferred with B cells from *Il21r*^*−/−*^ mice and WT controls. **D** Shown are the representative pictures for hematoxylin–eosin (HE) staining of liver/tumor tissues between the mice transferred with B cells from *Il21r*^*−/−*^ mice and WT controls. **E** Lipid droplets were significantly decreased in mice transferred with B cells from *Il21r*^*−/−*^ mice. Lipid droplets were quantified according to the image analysis of Oil Red O (ORO). Scale bar = 100μm in **D** and **E**. **F** Liver/tumor CD8^+^ T cells were significantly decreased in the mice transferred with B cells from *Il21r*^*−/−*^ mice. **G** Both effector (upper panel) and degranulating (lower panel) CD8^+^ T cells were significantly increased in the liver/tumor tissues of mice transferred with B cells from *Il21r*^*−/−*^ mice. **H** Liver/tumor IgA^+^ B cells were significantly decreased in *Il21r*^*−/−*^ mice. The representative flow cytometry images are shown in the upper panel. In the lower panel, the percentages of IgA^+^ B cells and the absolute cell number of IgA^+^ B cells per gram of liver/tumor tissues were analyzed between *Il21r*^*−/−*^ mice and wild type (WT) controls. **I** The level of serum IgA was significantly decreased in *Il21r*^*−/−*^ mice. The gating schemes are indicated above in **F**–**H**, and the number of mice in each group is shown in the panels accordingly. Student's *t* test was used to determine significance. * *P* < 0.05, ** *P* < 0.01, ns, not significant difference

IL-21R is expressed on a broad array of cell types including B cells and T cells. First, we checked which cells expressed IL-21R in MASH-driven HCC by using flow cytometry and immunofluorescence staining. Interestingly, we found that IL-21R was mostly expressed on CD19^+^ B cells (Fig. S9B, C). Subsequently, we applied an adoptive transfer experiment (Fig. 5A and Fig. S9D) to determine whether the B cells from *Il21r*^*−/−*^ mice had any effect on HCC tumorigenesis. Indeed, adoptive transfer of B cells from IL-21R-deficient mice into WT mice during the HCC stage suppressed tumorigenesis, accompanied by splenomegaly and decreased lipid accumulation, but no change for the liver weight (Fig. 5A-E). Even though the percentage of CD8^+^ T cells decreased, both effector and degranulating CD8^+^ T cells significantly increased after adoptive transfer with B cells from *Il21r*^*−/−*^ mice (Fig. 5F,G and Fig. S9E-H).

Next, we investigated how IL-21R affected B cells during MASH-driven HCC tumorigenesis. We noticed that the differential genes between *Il21r*^*−/−*^ mice and WT controls were also enriched in intestinal immune network for IgA production according to the KEGG pathway analysis (Fig. 3B). Given that IgA^+^ B cells were proven to be immunosuppressive cells in our previous work [17], we next checked whether IL-21R regulated the emergence of IgA^+^ B cells. Compared with WT controls, *Il21r*^*−/−*^ mice had significantly fewer liver and spleen IgA^+^ B cells (Fig. 5H and Fig. S10A, B). Moreover, the levels of serum IgA in *Il21r*^*−/−*^ mice were significantly decreased (Fig. 5I). These data indicate that IL-21R-deficient mice have fewer IgA^+^ B cells and thus impede MASH-driven HCC development.

**Fig. 6:**
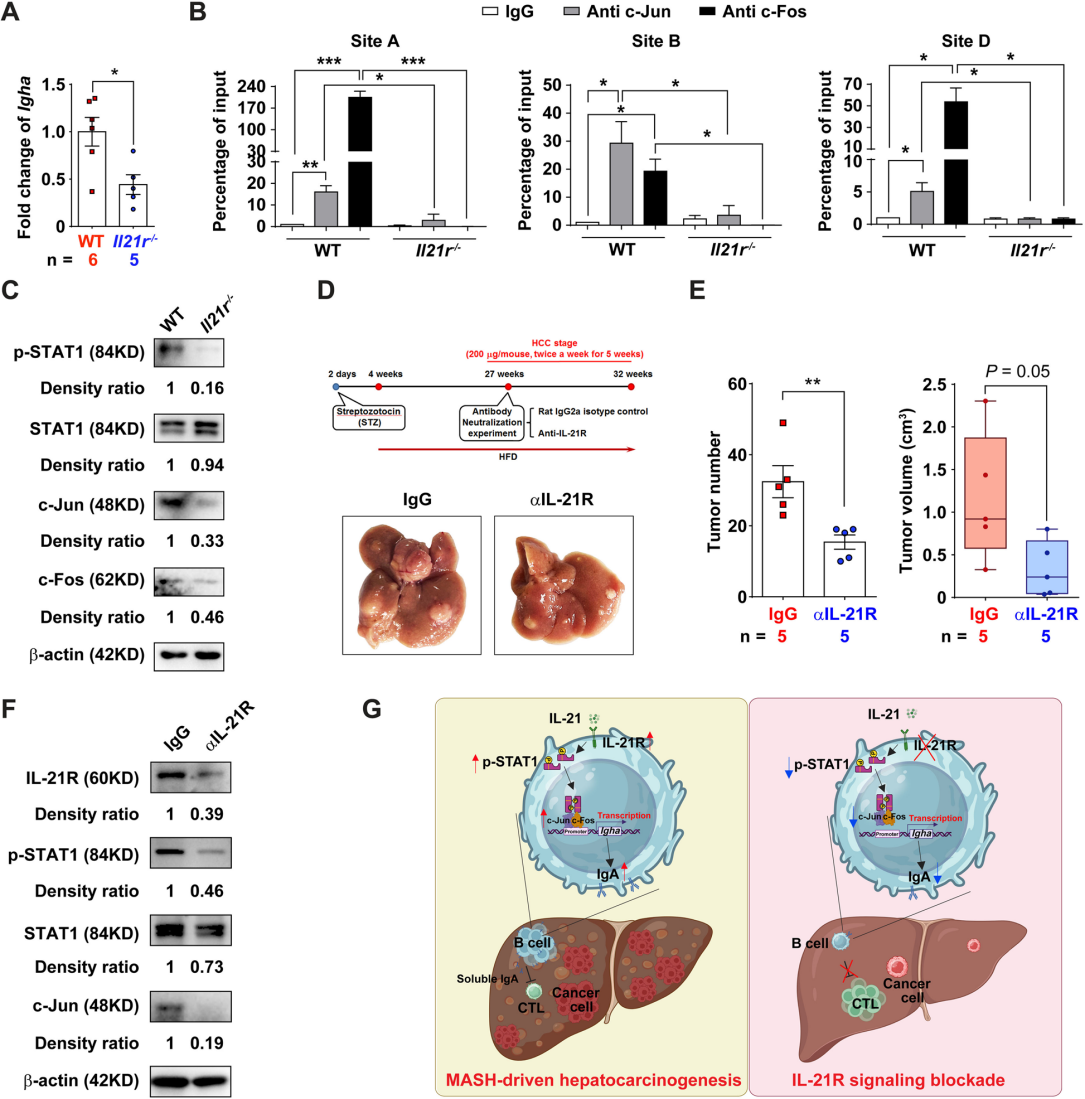
The IL-21R-STAT1-c-Jun/c-Fos-IgA axis and its implication in MASH-driven HCC. **A** The B cells isolated from *Il21r*^*−/−*^ mice exhibited lower *Igha* level compared to those isolated from wild type controls. Purified CD19^+^ B cells by using magnetic beads were obtained from the splenocytes of *Il21r*^*−/−*^ mice or wild type (WT) controls, and subsequently subjected to qRT-PCR to detect the mRNA level of *Igha*. **B** Ablation of IL-21R decreased the occupancy of c-Jun/c-Fos complex on the promoter of *Igha* in isolated B cells. The purified CD19^+^ B cells mentioned in A were subjected to chromatin immunoprecipitation. The chromatin complexes precipitated by the anti-c-Jun or anti-c-Fos antibody and the corresponding isotype-matched IgG were recovered and purified, and subsequently subjected to qRT-PCR using primer sets covering the AP-1 binding sites on the promoter of *Igha*. The binding sites are indicated above. **C** Ablation of IL-21R downregulated the protein levels of p-STAT1, c-Jun and c-Fos. The purified CD19^+^ B cells mentioned in A were subjected to western blotting. β-actin, internal control. **D** Shown are the schematic diagram of antibody neutralization experiment and the representative pictures of the endpoint liver from mice injected with IL-21R blocking antibody (αIL-21R) or its isotype control (IgG). **E** The number and volume of liver tumors were significantly decreased in the mice injected with IL-21R antibody compared to those injected with isotype control. **F** Blocking of IL-21R downregulated the protein levels of IL-21R, p-STAT1, STAT1 and c-Jun in the B cells. Purified CD19^+^ B cells by using magnetic beads were obtained from the splenocytes of mice injected with IL-21R blocking antibody or its isotype control, and subsequently subjected to western blotting. β-actin, internal control. **G** Model of IL-21R-STAT1-c-Jun/c-Fos-IgA axis and its implication in MASH-driven HCC. The number of mice in each group is shown in **A** and **E**. Student's *t* test was used to determine significance. * *P* < 0.05, ** *P* < 0.01, *** *P* < 0.001

To further explore the mechanism by which IL-21R regulated IgA^+^ B cells, we first confirmed that ablation of IL-21R decreased the mRNA level of *Igha* in B cells (Fig. 6A). We thus further evaluated the transcriptional regulation of *Igha*. Five consensus AP-1 binding sites were predicted (referred to Site A, B, C, D and E, respectively) between 2 kb upstream of the transcription start site (TSS) and the TSS (Fig. S11A). AP-1 is an inducible transcription factor complex consisting of a group of transcription factors including Jun, Fos and ATF family proteins. ChIP assay confirmed the existence of a direct interaction between c-Jun/c-Fos and Site A, B, D of the *Igha* promoter in B cells from WT controls, but not in B cells from *Il21r*^*−/−*^ mice (Fig. 6B), suggesting that c-Jun/c-Fos modulates the transcription of *Igha* by binding to the Site A, B, D. IL-21 has been shown to activate the JAK-STAT pathway, and STAT1 can interact with AP-1 to trigger transcriptional activation [37]. Thereafter, we further showed that IL-21R, phosphorylated STAT1, STAT1 and c-Jun were upregulated in B cells during MASH-driven heptocacinogenesis (Fig. S11B), and ablation of IL-21R decreased the protein levels of phosphorylated STAT1, c-Jun and c-Fos (Fig. 6C). Furthermore, STAT1 inhibitor blocked the elevation of c-Jun without change the expression of IL-21R, resulting in the downregulation of *Igha* mRNA level in B cells from mice at HCC stage (Fig. S11C, D). Importantly, blockade of IL-21R signaling with an IL-21R blocking antibody induced tumor regression by enhancing cytotoxic CTL activation (Fig. 6D-F and Fig. S12).

Taken together, we suggest an IL-21R-STAT1-c-Jun/c-Fos-IgA regulatory pathway (Fig. 6G): IL-21R is increased in MASH-driven HCC, resulting in upregulation of phosphorylated STAT1, which acts as a co-activator of c-Jun/c-Fos. The c-Jun/c-Fos complex binds to the promoter of *Igha* and promotes the transcription of *Igha*, resulting in the emergence of immunosuppressive IgA^+^ B cells, and thereby inhibits CTL activation and eventually facilitates MASH-driven HCC. Importantly, ablation of IL-21R can reduce the induction of IgA^+^ B cells, and thus enhances CTL activation and impedes MASH-driven HCC. These findings indicate that IL-21R promotes the tumorigenesis of MASH-driven HCC by inducing immunosuppressive IgA^+^ B cells and disrupting the IL-21R regulatory axis may be beneficial for MASH-driven HCC therapy.

## Discussion

In the present study, we revealed that the IL-21R-STAT1-c-Jun/c-Fos-IgA regulatory pathway was activated during MASH-driven hepatocarcinogenesis, leading to the induction of immunosuppressive IgA^+^ B cells, and thereby inhibited CTL activation and eventually facilitated MASH-driven HCC in vivo. These findings support the immunosuppressive effect of IL-21/IL-21R in tumorigenesis. To our knowledge, this is the first attempt to circumstantiate the function of IL-21R in MASH-driven hepatocarcinogenesis, and to elucidate the mechanism by which IL-21R induces IgA^+^ B cells.

IL-21/IL-21R signaling has been implicated in the regulation of inflammation in various acute and chronic inflammatory diseases. The role of IL-21/IL-21R in cancer development remains obscure and has not been extensively investigated in faithful in vivo models. To date, the function and mechanism of IL-21/IL-21R during MASH-driven hepatocarcinogenesis have not yet been reported. Here, we presented the following evidences: (i) Assessment of the levels of IL-21R by using IHC staining revealed that elevated IL21R expression was correlated with advanced stage of HCC and poor survival in our cohort (*n* = 69), and the conclusion was validated in two additional independent cohorts (*n* = 80 and 365, respectively) according to the TCGA database. Importantly, HCC patients with high IL-21R expression exhibited severe steatosis. (ii) Both the mRNA and protein levels of IL-21R were elevated in liver tumors in a well-established mouse model of MASH-driven HCC, the STAM model (*n* = 52 in total). (iii) By using two different in vivo mouse models of MASH-driven HCC, the STAM model and the WD&High sugar solution&CCl_4_ model, we revealed that ablation of IL-21R obstructed MASH-driven HCC by enhancing CTL activation with large number of mice (*n* = 86 in total). (iv) Adoptive transfer experiment (*n* = 14 in total) demonstrated that adoptive transfer of B cells from *Il21r*^*−/−*^ mice into wild type mice during the HCC stage suppressed MASH-driven hepatocarcinogenesis in recipient mice. (v) According to the results of flow cytometry, ELISA, western blotting and ChIP assay, we elucidated that ablation of IL-21R limited the induction of immunosuppressive IgA^+^ B cells via the IL-21R-STAT-1-c-Jun/c-Fos-IgA regulatory axis. (vi) Blockade of IL-21R signaling with an IL-21R blocking antibody induced tumor regression of MASH-driven HCC. Collectively, our data suggest that, perhaps in MASH-driven HCC, IL-21R plays a pro-tumorigenic inflammatory role and that blockade of IL-21R signaling will result in attenuated MASH-driven HCC development.

IL-21 was originally demonstrated to be a growth and survival factor in human myeloma cell lines, which was mediated through the activation of JAK1/STAT3 signaling [21]. However, since then, a large number of reports have shown that IL-21 promotes tumor clearance, rather than tumor survival [19]. Subsequently, the discovery of the immunosuppressive actions of IL-21 further suggested that IL-21 is a “double-edged sword”: IL-21 stimulation may lead to either the induction or suppression of immune responses [18]. Thus, both the stimulatory and suppressive effects of IL-21 must be considered during the clinical use of IL-21-related immunotherapeutic agents. Moreover, the biological effects of IL-21 are also influenced by the presence of other cytokines or signaling molecules in the tumor microenvironment [18]. As the private receptor of IL-21, IL-21R can also be either immunosuppressive or immunostimulatory depending on the environmental context. To date, only one publication attempted to address the anti-tumor role of IL-21R in HCC development [24], particularly utilizing xenograft mouse models. Since such models have clear advantages in terms of rapid and uniform tumor growth, they may be less perfect for studying mechanisms which rely on a complicated tumor microenvironment and chronic inflammation, a key component regulated by cytokines in cancer. In contrast to their conclusion, our study demonstrated that ablation of IL-21R impeded liver cancer development, particularly MASH-driven HCC, in two different mouse models of spontaneous MASH-driven HCC: the STAM model and the WD&High sugar solution&CCl_4_ model. Along with human data on poor survival, advanced tumor stages and severe steatosis in HCC patients with high IL21R expression, our work implies that IL-21R plays a cancer-promoting role in MASH-driven HCC. An essential component of this mechanism is mediated by immunosuppressive IgA^+^ B cells via the IL-21R-STAT-1-c-Jun/c-Fos-IgA regulatory axis.

B cells are well known as key mediators of humoral immune responses via the production of antibodies, and immunoglobulin A is one of the most abundantly produced antibody isotypes. It has been reported that immunoglobulin A and IgA^+^ B cells exert anti- or pro- tumor effect in different tumor types [17, 38–42]. Elevated levels of intratumoral IgA have been shown to be associated with poor outcomes in patients with bladder cancer [38], colorectal cancer [39] and melanoma [40]. Additionally, our colleagues discovered that IgA^+^ B cells, which expressed PD-L1, IL-10 and Fas-L, could suppress anti-tumor immunity in oxaliplatin-treated pancreatic cancer [41], one of the mechanisms responsible for chemotherapy tolerance. Thereafter, together with our colleague, we demonstrated that inflammation-induced immunosuppressive IgA^+^ B cells dismantle anti-cancer immunity by suppressing CTL activation during MASH-driven HCC [17]. Although there is more than sufficient evidence indicating that IgA^+^ B cells exert prominent immunosuppressive effects, the anti-tumor effect of IgA^+^ B cells has also been reported. For instance, in ovarian cancer, tumor antigen-specific and tumor antigen-independent IgA responses antagonized the growth of tumor by governing coordinated tumor cell, T cell and B cell responses [42]. With respect to the reasons for the induction of IgA^+^ B cells, emerging evidence indicates that a combination of host, environmental and tumor factors mediates IgA class switching through upregulating class switching-related genes. First, TGF-β, which is highly expressed in the tumor microenvironment, can activate the constant heavy chain α (Cα) gene promoter to trigger T-cell-independent IgA class switching [43, 44]. Second, fatty acids or microbial products can activate Toll like receptors, which promote the activation of IκB kinase (IKK) complex to trigger the translocation of IκB-free NF-κB to the nucleus, and initiate IgA class switching by binding to the activation-induced cytidine deaminase (AID) gene promoter [44–46]. Nevertheless, no specific transcriptional markers have been identified to exclusively define the IgA^+^ B cells.

It is thought that IL-21/IL-21R plays a pivotal role in IgA production. When combined with IL-4 and anti-CD40, IL-21 can induce class switch recombination to IgA and differentiation of IgA^+^ B cells in human peripheral blood mononuclear cells (PBMCs) [47]. Additionally, IL-21 ensures TGF-β-induced IgA isotype expression in mouse Peyer’s patches [27]. Moreover, it’s widely explored that IL-21 promotes intestinal IgA^+^ B cell production [28–31], possibly through upregulating differentiation-related and class switching–related genes such as *Aicda*, *Ski* and *Prdm1* [30, 31]. Additionally, in the presence of atypical commensals such as segmented filamentous bacteria and *Helicobacter*, IL-21R-deficient mice exhibit reduced IgA^+^ B cells in the germinal center, Peyer’s patches and small intestine [29]. However, the relationship between IL-21/IL-21R and IgA in liver or tumor microenvironment is unclear. Consist with the mouse [29] and human clinical data [48], here we presented that IL-21R-deficient mice exhibited reduced numbers of spleen or liver/tumor IgA^+^ B cells and soluble IgA during MASH-driven hepatocarcinogenesis. Importantly, we elucidated the mechanism in which IL-21R activated IgA via the IL-21R-STAT-1-c-Jun/c-Fos-IgA regulatory axis. Moreover, we demonstrated that AP-1, mainly composed of c-Jun and c-Fos, was the right transcription factor for identifying IgA^+^ B cells.

To our knowledge, the current study provides the most comprehensive investigation regarding the cancer-promoting role of IL-21R by exerting immunosuppressive characteristics of B cells in MASH-driven hepatocarcinogenesis to date, implying that targeting IL-21R signaling represents a potential therapeutic strategy for cancer therapy. However, our study is not without limitations. Firstly, B cell-specific knockout of IL-21R mice cannot be generated by crossing *Il21r* ^flox/flox^ mice with CD19-cre or Mb1-cre mice in this study because all of these genes are located on chromosome seven. Secondly, there are no gross defects in the development of the immune system in *Il21r*^*−/−*^ mice [49] or human [48]. However, certain patients with loss-of-function mutations in the IL-21R gene suffer from recurrent respiratory and gastrointestinal infections, additionally have cryptosporidiosis, leading to secondary cholangitis and liver disease according to some case reports [48, 50, 51]. Therefore, precision therapy based on IL-21R deficiency should be considered in both experiment and clinic in the future. Thirdly, according to our IHC staining result and the data from The Human Protein Atlas (https://www.proteinatlas.org/), IL-21R also expressed in the cytoplasma and membrane of tumor cells in human liver cancer. The role and mechanism of IL-21R in HCC cells are still yet to be explored.

## Conclusions

In conclusion, our findings explore the cancer-promoting role of IL-21R in MASH-driven hepatocarcinogenesis and elucidate the mechanism by which IL-21R activates IgA via the IL-21R-STAT-1-c-Jun/c-Fos-IgA regulatory axis. Thus, targeting IL-21R signaling represents a potential therapeutic strategy for cancer therapy.

### Supplementary Information

**Supplementary Material 1.**

**Supplementary Material 2.**

**Supplementary Material 3.**

**Supplementary Material 4.**

## Data Availability

Whole transcriptome sequencing data in this study are available in the Sequence Read Archive (SRA) database under accession number PRJNA1035974. All data supporting the conclusions of this article are included within the article and its additional files. Any other information is available from the corresponding authors upon reasonable request.

## Abbreviations

HCC: Hepatocellular carcinoma
MASLD: Metabolic dysfunction-associated steatotic liver disease
MASH: Metabolic dysfunction-associated steatohepatitis
NASH: Non-alcoholic steatohepatitis
NAFLD: Non-alcoholic fatty liver disease
IL-21: Interleukin-21
IL-21R: Interleukin-21 receptor
CTL: CD8^+^ T lymphocyte
JAK: Janus kinase
STAT: Signal transducers and activators of transcription
AP-1: Activating protein 1
TSS: Transcription start site
HBV: Hepatitis B virus
HCV: Hepatitis C virus
HFD: High-fat diet
WD: Western diet
DMEM: Dulbecco's modified Eagle's medium
FBS: Fetal bovine serum
PBS: Phosphate-buffered saline
qRT-PCR: Real time quantitative RT-PCR
HE: Haematoxylin and eosin
IHC: Immunohistochemistry
IF: Immunofluorescent
SR: Sirius Red
ORO: Oil Red O
ChIP: Chromatin immunoprecipitation
ELISA: Enzyme-linked immunosorbent assay
GO: Gene Ontology
KEGG: Kyoto Encyclopedia of Genes and Genomes
GSEA: Gene Set Enrichment Analysis
SEM: Standard error of the mean
ANOVA: Analysis of variance
