# Supplementary material for: Interleukin-21 receptor signaling promotes metabolic dysfunction-associated steatohepatitis-driven hepatocellular carcinoma by inducing immunosuppressive IgA+ B cells
Source: Mol Cancer. 2024 May 8;23:95. doi: 10.1186/s12943-024-02001-2 (PMC11077880; doi:10.1186/s12943-024-02001-2)
Supplement: Supplementary file 1 — Supplementary Material 1. [file 12943_2024_2001_MOESM1_ESM.pdf]

Supplementary Materials and Methods

The Cancer Genome Atlas (TCGA) database analysis

For relapse-free survival analysis of interleukin-21 receptor (IL-21R) in male hepatocellular carcinoma (HCC) patients without hepatitis B virus (HBV), the corresponding results were analyzed and downloaded from Kaplan–Meier Plotter (https://kmplot.com/analysis/)[1]. For overall survival analysis of IL-21R in HCC patients, the related data was acquired manually from The Human Protein Atlas (https://www.proteinatlas.org/) and summarized in Supplementary Table S5. The “minimum *P*-value” was applied to obtain the most appropriate cut-off for separating the IL-21R-low from the IL-21R-high expression group of patients. Kaplan–Meier survival curve was then constructed using SPSS software (Chicago, IL, USA).

Whole transcriptome sequencing processing and analysis

For long non-coding RNA (lncRNA) library preparation, the RNA was treated with RNase H kit to remove ribosomal RNA (rRNA), followed by RNA fragmentation reaction. Thereafter, the first-strand and the second-strand cDNAs were sequentially synthesized by using pre-prepared first-strand synthesis reaction mixture and second-strand synthesis reaction mixture containing dUTP, respectively. Next, double-stranded cDNA fragments were subjected to end-repair, and then a single ‘A’ nucleotide was added to the 3’ ends of the blunt fragments. The adaptors were subsequently ligated to the cDNAs, followed by PCR amplification. For small RNA library preparation, the RNA was sequentially ligated with 3’ and 5’ adaptors. Thereafter, reverse transcription reaction was applied and the PCR products were purified with PAGE gel.

Either the PCR products for lncRNA library or the purified PCR products for small RNA library were denatured into single-stranded DNA for circularization, while the uncyclized linear DNA molecules were digested. Single-stranded cyclized products were further replicated via rolling cycle amplification, resulting in a DNA nanoball (DNB) containing multiple copies of DNA. Sufficient quality DNBs were subsequently loaded into patterned nanoarrays by using high-intensity DNA nanochip technique and sequenced through combinatorial Probe-Anchor Synthesis (cPAS).

The sequencing data from lncRNA library was filtered with SOAPnuke[2] by several steps: (1) Removing reads containing sequencing adapter; (2) Removing reads whose low-quality base ratio (base quality less than or equal to 15) is more than 20%; (3) Removing reads whose unknown base ('N' base) ratio is more than 5%. Thereafter, the clean reads were mapped to the mouse genome (GRCm38/mm10) using HISAT2[3] for messenger RNA (mRNA) and lncRNA, or applied to CircBase to annotate circular RNA (circRNA). After that, the expression levels of mRNA and lncRNA were calculated by RSEM (v1.3.1)[4], while the expression level of circRNA was calculated using reads spanning the junction site (the head-to-tail junction of the circRNA sequence) with at least 10 bp coverage at both ends.

The sequencing data from small RNA library was also filtered with SOAPnuke [2] by several steps: (1) Removing low quality tags; (2) Removing tags with 5’ adaptor contaminants; (3) Removing tags without 3’ adaptor; (4) Removing tags without insertion; (5) Removing tags with poly A; (6) Removing tags shorter than 15nt.

The data analysis was further performed on Dr. Tom Multi-omics Data Mining System

(https://biosys.bgi.com).

Flow cytometry

For lymphocyte isolation, livers/tumor tissues were cut into small pieces and incubated at 37 ℃ for 30 minutes with gentle shaking at 200 rpm in the following digestion buffer: DMEM medium supplemented with 5% fetal bovine serum (FBS), 1 mg/mL collagenase type IV (#C5138, Sigma-Aldrich, St. Louis, MO, USA) and 20 μg/mL DNase I (#DN25, Sigma-Aldrich, St. Louis, MO, USA). For splenocyte isolation, spleens were gently smashed. After incubation or smash, cell suspensions were passed through a 100 μm cell strainer and washed with 1 × phosphate-buffered saline (PBS) containing 2% FBS and 2 mM EDTA. Isolated cells were stained with labelled surface antibodies in 1 × PBS containing 2 mM EDTA. Dead cells were excluded on the basis of staining with Live/Dead Fixable Viability Dye (FVD-eFluor780, #65-0865-14, Invitrogen, Carlsbad, CA, USA). For intracellular cytokine staining, cells were stimulated with cell stimulation cocktail containing PMA (#S1819, Beyotime, Shanghai, China) and ionomycin (#S1672, Beyotime, Shanghai, China), in the presence of Golgi stop (#51-2092KZ, BD Biosciences, San Diego, CA, USA). Particularly, the CD107a staining antibody was added to the culture during the stimulation for CD107a measurement. After four hours stimulation, cells were fixed and permeabilized either with BD Cytofix/Cytoperm reagent (#51-2091KZ, BD Biosciences, San Diego, CA, USA) for cytokine staining, or Foxp3/Transcription Factor staining buffer (#00-5523-00, Invitrogen, Carlsbad, CA, USA) for combined staining of cytokines and transcription factors. After fixation/permeabilization, cells were stained with labelled antibodies of interest. Cells were analyzed on BD FACS Verse Flow Cytometer (BD Biosciences, San Jose, CA, USA) and the

data were analyzed using FlowJo X 10.0.7 software (Tree Star, Ashland, OR, USA). Absolute numbers of particular immune cells in spleen or livers/tumor tissues were calculated as described previously[5]. The antibodies used for flow cytometry are provided in Table S2.

Histology

For paraffin-embedded tissue section, livers were fixed in 10% neutral-buffered formalin and embedded in paraffin. Sections were subsequently prepared and stained with haematoxylin (#G1004, Servicebio, Wuhan, China) and eosin (#G1001, Servicebio, Wuhan, China), Sirius Red (#G1018, Servicebio, Wuhan, China), or processed for immunohistochemistry (IHC) staining. For frozen section, livers were embedded in OCT compound (#4583, Tissue-Tek, Sakura Finetek, Torrance, CA, USA) and frozen, sectioned and stained with Oil Red O (#G1015, Servicebio, Wuhan, China) and haematoxylin. Five random fields (50×), one representative field (100×) and five random fields (200×) were screened for Sirius Red analysis, Oil Red O analysis and IHC staining analysis, respectively. The images were further quantitated by using Image J software (NIH, Maryland, USA) to evaluate collagen deposition, lipid accumulation and positive cell.

Enzyme-linked immunosorbent assay (ELISA) and colorimetric protein detection

Mouse serum IgA (#ARG81183, Arigo Biolaboratories Corp, Hsinchu, Taiwan), serum ALT (#1007-00-03-06, Rsbio, Shanghai, China), triglyceride (#BC0625, Solarbio, Beijing, China), total cholesterol (#BC1985, Solarbio, Beijing, China) and free cholesterol (#BC1895, Solarbio, Beijing, China) were measured according to manufacturer's instructions, respectively.

Real-time quantitative reverse transcriptase-polymerase chain reaction (qRT-PCR) analysis

Total RNA was extracted using the TRI reagent (#T9424, Sigma-Aldrich, MO, USA). The levels of target genes were normalized against the level of reference gene (*Actb*) by $2^{-\Delta Ct}$, where $\Delta Ct = Ct_{target} - Ct_{reference}$. The primer sequences used for qRT-PCR are listed in Supplementary Table S3.

Western blotting

Mouse liver/tumor extracts or cell lysates were separated in a SDS-polyacrylamide gel, electrophoretically transferred to a PVDF membrane (Roche Diagnostics, Mannheim, Germany), and incubated sequentially with primary and secondary antibodies. The signal was developed with a commercial ECL kit (Millipore, Billerica, MA, USA). The antibodies used for western blotting are listed in Supplementary Table S2.

Immunohistochemistry staining

Paraffin-embedded tissue sections were processed for antigen retrieval by microwave heating in 10 mM citrate buffer (pH 6.0), and immunostained with primary antibody against human or mouse IL-21R, mouse F4/80, mouse Ki67 at 4 ℃ overnight. Next, the sections were incubated with corresponding HRP conjugates and developed with DAB substrate solution. Thereafter, the sections were counterstained with hematoxylin, and mounted in neutral resin. Specially, to evaluate human IL-21R expression, the whole tissue sections were scanned under a microscope. The intensity score was defined as negative (0), weak (1), medium (2) or strong (3) staining. The fraction score was then calculated based on the proportion of IL-21R-stained cells, which was divided into 0% (0), 1-25% (1), 25-50% (2), 50-75% (3) and

75-100% (4). The intensity and fraction scores were multiplied to obtain a total score, which ranged from 0 to 12 and represented the expression levels of IL-21R. The antibodies used for immunohistochemistry staining are listed in Supplementary Table S2.

Immunofluorescent staining

Frozen sections were warmed up at room temperature for 30 minutes. To eliminate autofluorescence, the sections were first incubated with tissue autofluorescence quencher solution (#G1221, Servicebio, Wuhan, China) at room temperature for 30 minutes. The sections were further incubated with 3% bovine serum albumin (BSA) for one hour to block unspecific binding of the antibodies, followed by incubation with the mixture of two primary antibodies at 4 ℃ overnight. Thereafter, the sections were incubated with the mixture of Alexa Fluor-conjugated secondary antibodies at room temperature for one hour. Subsequently, the sections were counterstained with 4'-6'-diamidino-2-phenylindole (DAPI) staining solution (#abs47047616, Absin, Shanghai, China) for four minutes, and then mounted in mounting medium. The antibodies used for immunofluorescent staining are listed in Supplementary Table S2.

Terminal deoxynucleotidyl transferase-mediated dUTP nick end labeling (TUNEL) assay

TUNEL assay (#G1507, Servicebio, Wuhan, China) were performed according to manufacturer's instructions. For analysis, five random fields (200×) were screened and further quantitated by using Image J software (NIH, Maryland, USA) to evaluate the apoptotic cell death.

Glucose tolerance test

Mice were transferred to clean cages with no food or feces in the hopper or the bottom of the cage and fasted overnight with access to drinking water. Small drops of blood were obtained from tail cuts and assessed for baseline glucose levels using a One-touch Ultra 2 glucometer (Lifescan, Johnson & Johnson). A 10% glucose solution was intraperitoneally injected (1 g/kg mouse body mass). Small drops of blood was sampled at 30, 60, 120 min, and detected for glucose levels.

Reference

[1] Gyorffy B. Survival analysis across the entire transcriptome identifies biomarkers with the highest prognostic power in breast cancer. Comput Struct Biotechnol J 2021;19: 4101-4109.

[2] Li R, Li Y, Kristiansen K, et al. SOAP: short oligonucleotide alignment program. Bioinformatics 2008;24(5):713-714.

[3] Kim D, Langmead B, Salzberg SL. HISAT: a fast spliced aligner with low memory requirements. Nat Methods 2015;12(4): 357-360.

[4] Li B, Dewey CN. RSEM: accurate transcript quantification from RNA-Seq data with or without a reference genome. BMC Bioinformatics 2011;12: 323.

[5] Shalapour S, Lin XJ, Bastian IN, et al. Inflammation-induced IgA^+ cells dismantle anti-liver cancer immunity. Nature 2017;551:340-345.
