# Supplementary material for: Interleukin-21 receptor signaling promotes metabolic dysfunction-associated steatohepatitis-driven hepatocellular carcinoma by inducing immunosuppressive IgA+ B cells
Source: Mol Cancer. 2024 May 8;23:95. doi: 10.1186/s12943-024-02001-2 (PMC11077880; doi:10.1186/s12943-024-02001-2)
Supplement: Supplementary file 2 — Supplementary Material 2. [file 12943_2024_2001_MOESM2_ESM.pdf]

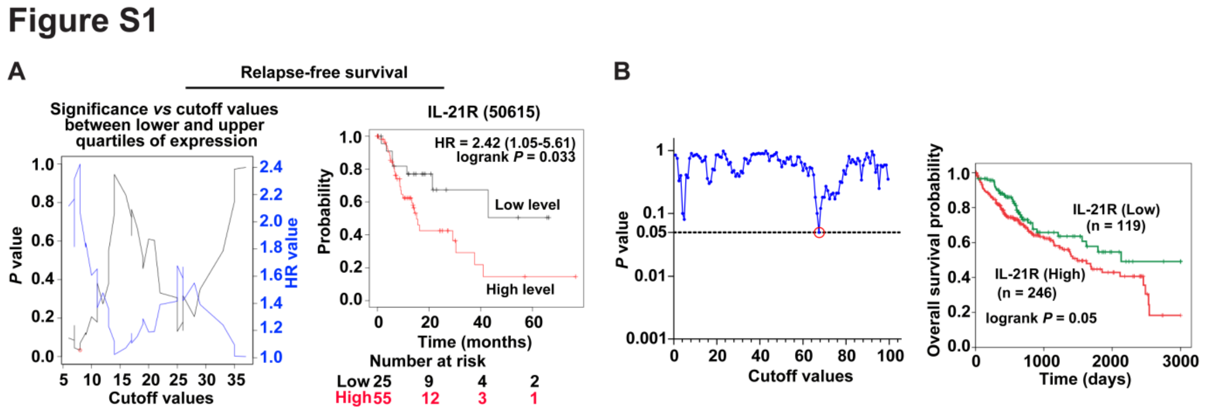

Figure S1. Higher expression of IL-21R is associated with shorter survival in hepatocellular carcinoma patients. (**A**) Higher mRNA level of IL-21R was associated with shorter relapse-free survival in male HCC patients without HBV (n = 80) according to Kaplan–Meier Plotter. (**B**) Higher mRNA level of IL-21R was associated with shorter overall survival in HCC patients. Minimum *P*-value was acquired by log-rank survival analysis using a series of percentile values as cutoff points, and the 67.4th percentile was the most appropriate for separating the IL-21R-low from the IL-21R-high level group (left panel). Kaplan-Meier overall survival analysis for HCC patients (n = 365) was then conducted according to the mRNA level of IL-21R (right panel).

Figure S2

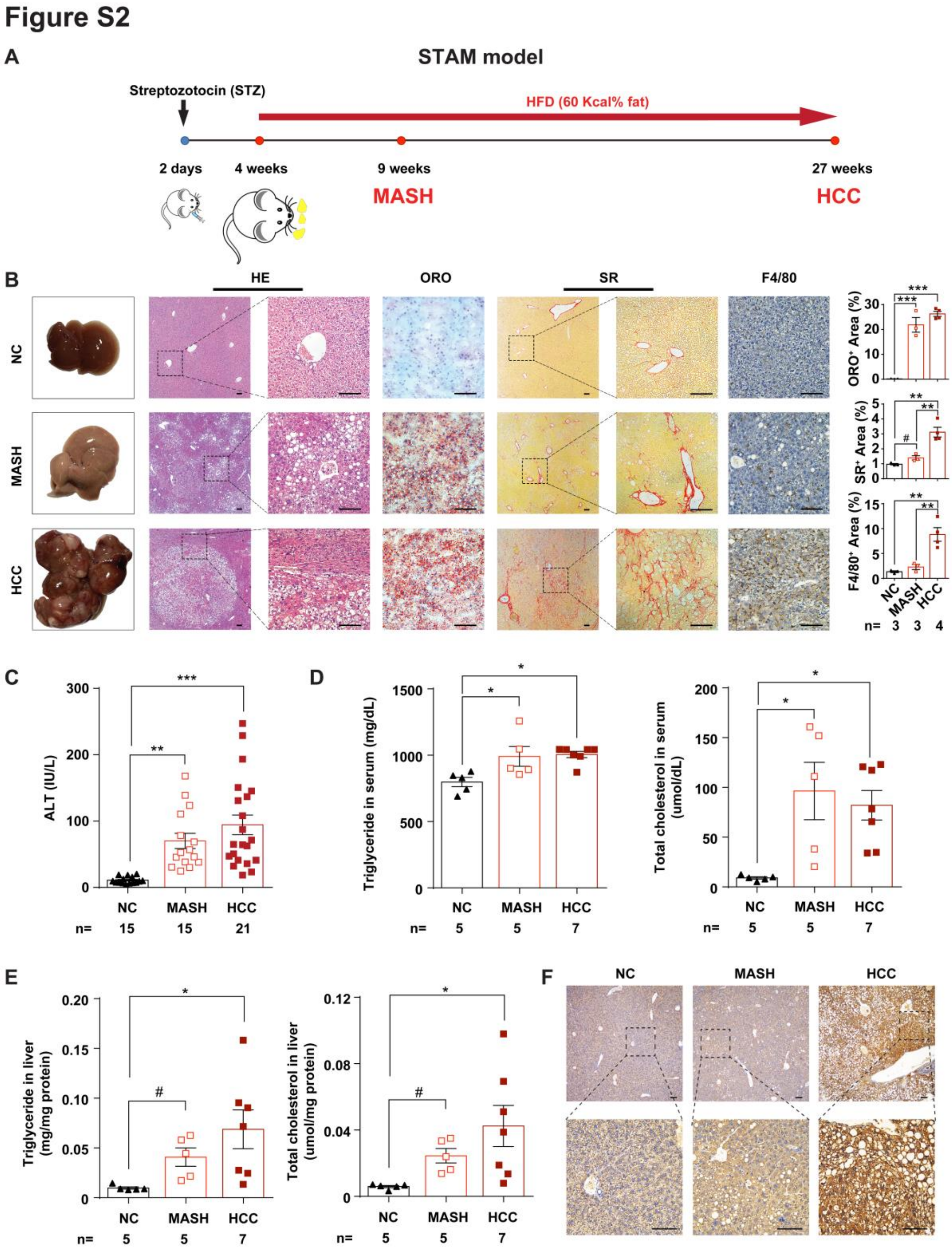

Figure S2. Establishment and validation of STAM model, which is a metabolic dysfunction-associated steatohepatitis-driven hepatocellular carcinoma mouse model. (**A**) Schematic diagram of STAM model. HFD, high fat diet. MASH, metabolic dysfunction-associated steatohepatitis. HCC, hepatocellular carcinoma. (**B**) Steatosis, fibrosis

and infiltration of inflammatory cells occurred in the liver of mice with MASH or HCC. Shown are the representative pictures for hematoxylin-eosin (HE), Oil Red O (ORO), Sirius Red (SR) and F4/80 immunohistochemistry staining of the liver tissues from mice at normal chow (NC), MASH and HCC stages. (**C**) Liver damage occurred in the mice with MASH or HCC. Serum ALT levels were detected in the mice at the above-mentioned stages. Note that the data for HCC group is the same as those for WT group in Figure 2C. (**D, E**) Triglyceride and total cholesterol levels were increased in the serum or liver/tumor extracts of mice with MASH or HCC. Serum (D) or liver/tumor extracts (E) were analyzed for triglyceride and total cholesterol. (**F**) The expression level of IL-21R was upregulated in MASH-driven HCC. The expression of IL-21R was detected in the liver tissues from mice at the above-mentioned stages by using immunohistochemistry staining. Scale bar = 100 μm. The number of mice in each group is shown in the panels accordingly. One-way ANOVA was used to determine significance. # All were significant using Student's *t* test. * $P < 0.05$, ** $P < 0.01$, *** $P < 0.001$.

Figure S3

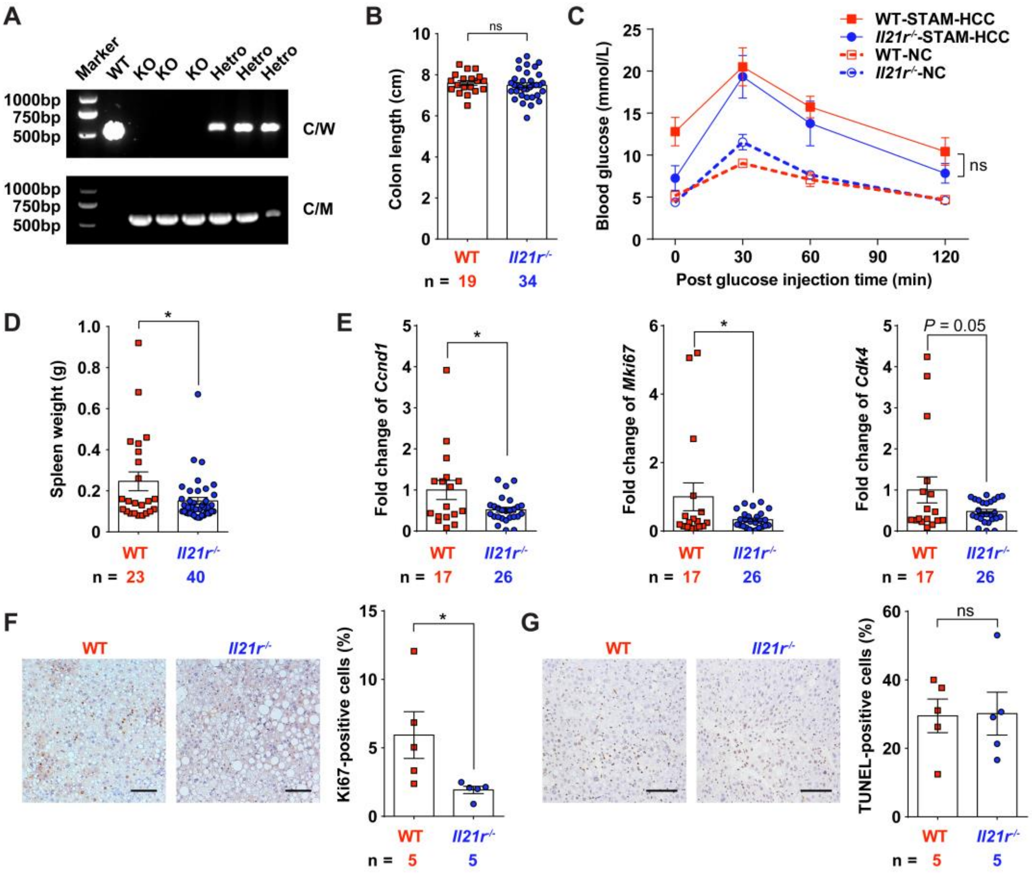

Figure S3. The comparison of colon length, glucose tolerance, spleen weight, proliferation markers and apoptotic cell death between *Il21r*$^{-/-}$ mice and wild type controls at 27 weeks of age in the STAM model. (**A**) The genotyping validation for *Il21r*$^{-/-}$ mice. WT, wild type control; KO, *Il21r* total knock-out mice; Hetro, hetrozyotes. C/W, detection for wild type allele band; C/M, detection for mutant allele band. (**B**) Ablation of IL-21R did not change the colon length of mice. (**C**) Glucose tolerance was not different between *Il21r*$^{-/-}$ mice and WT controls in the STAM model or normal chow diet. (**D**) The weight of spleen was significantly decreased in *Il21r*$^{-/-}$ mice. (**E**) The expression levels of proliferation markers were decreased in *Il21r*$^{-/-}$ mice. (**F**) The Ki67-positive cells were decreased in the tumor tissues of *Il21r*$^{-/-}$ mice. (**G**) Ablation of IL-21R did not change the apoptotic cell death in the tumor tissues. Scale bar = 100 μm. The number of mice in each

group is shown in the panels accordingly. Two-way ANOVA and Student's *t* test were used to determine significance in C and other panels, respectively. * $P < 0.05$, ns, not significant difference.

Figure S4

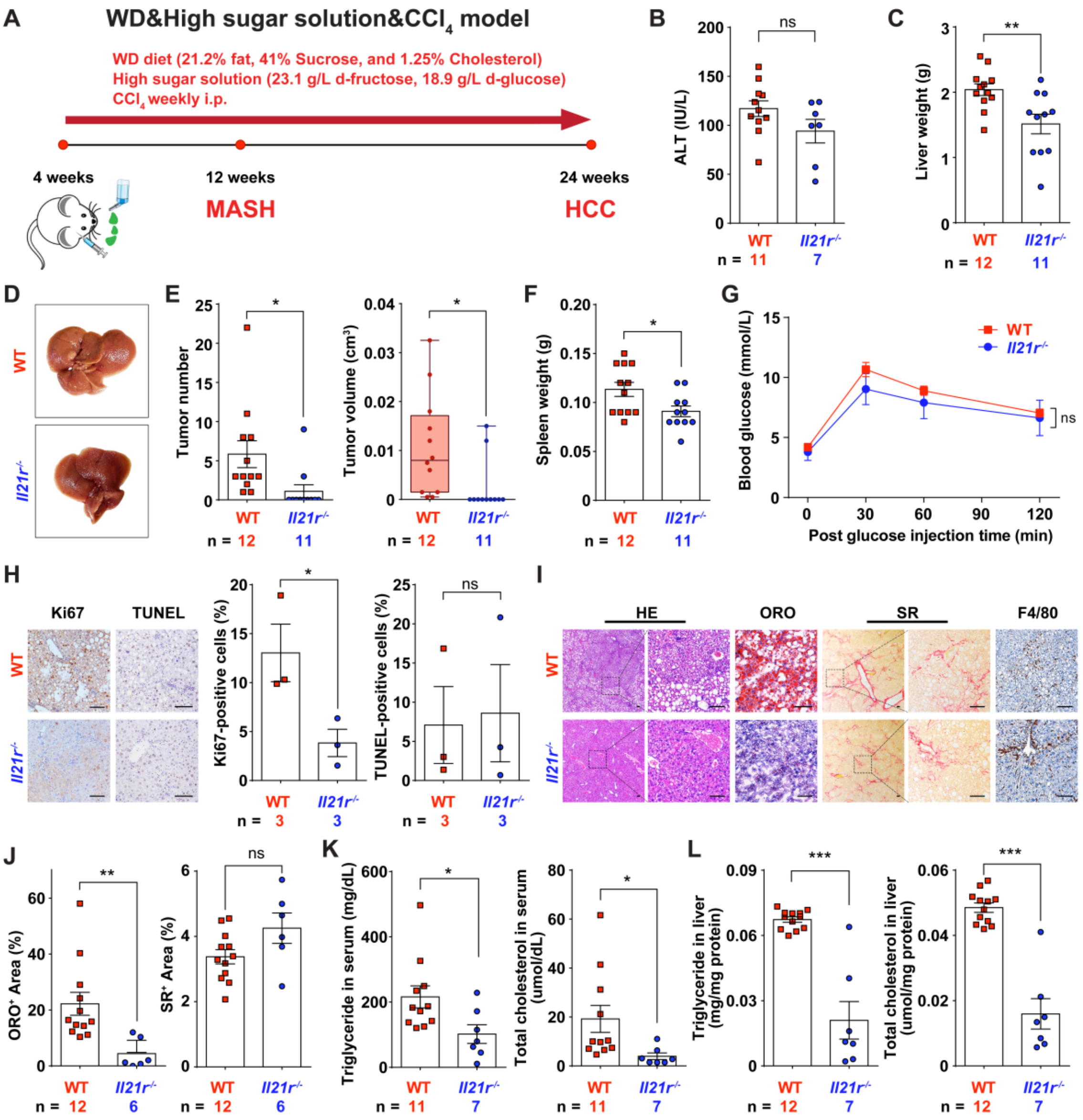

Figure S4. *Il21r*$^{-/-}$ mice exhibit less tumor burden of HCC with decreased lipid droplets in the WD&High sugar solution&CCl_4 model. (**A**) Schematic diagram of WD&High sugar solution&CCl_4 model. WD, western diet. MASH, metabolic dysfunction-associated steatohepatitis. HCC, hepatocellular carcinoma. (**B-G**) Comparison of serum ALT level (B),

liver weight (C), representative pictures of liver (D), the number and volume of tumors (E), spleen weight (F) and glucose tolerance (G) between *Il21r*$^{-/-}$ mice and wild type (WT) controls in the WD&High sugar solution&CCl_4 model. (**H**) Ablation of IL-21R decreased the proliferative capacity but did not change the apoptotic cell death in the WD&High sugar solution&CCl_4 model. (**I**) Shown are the representative pictures for hematoxylin-eosin (HE), Oil Red O (ORO), Sirius Red (SR) and F4/80 immunohistochemistry staining of the liver/tumor tissues between *Il21r*$^{-/-}$ mice and WT controls in the WD&High sugar solution&CCl_4 model. (**J**) Lipid droplets were significantly decreased in *Il21r*$^{-/-}$ mice. Lipid droplets and collagen deposition were quantified according to the image analysis of ORO and SR staining, respectively. (**K, L**) Triglyceride and cholesterol were significantly decreased in the serum or liver/tumor extracts of *Il21r*$^{-/-}$ mice. Serum (K) or liver/tumor extracts (L) were analyzed for triglyceride and total cholesterol. Scale bar = 100 μm. The number of mice in each group is shown in the panels accordingly. Two-way ANOVA and Student's *t* test were used to determine significance in G and other panels, respectively. * $P < 0.05$, ** $P < 0.01$, *** $P < 0.001$, ns, not significant difference.

Figure S5

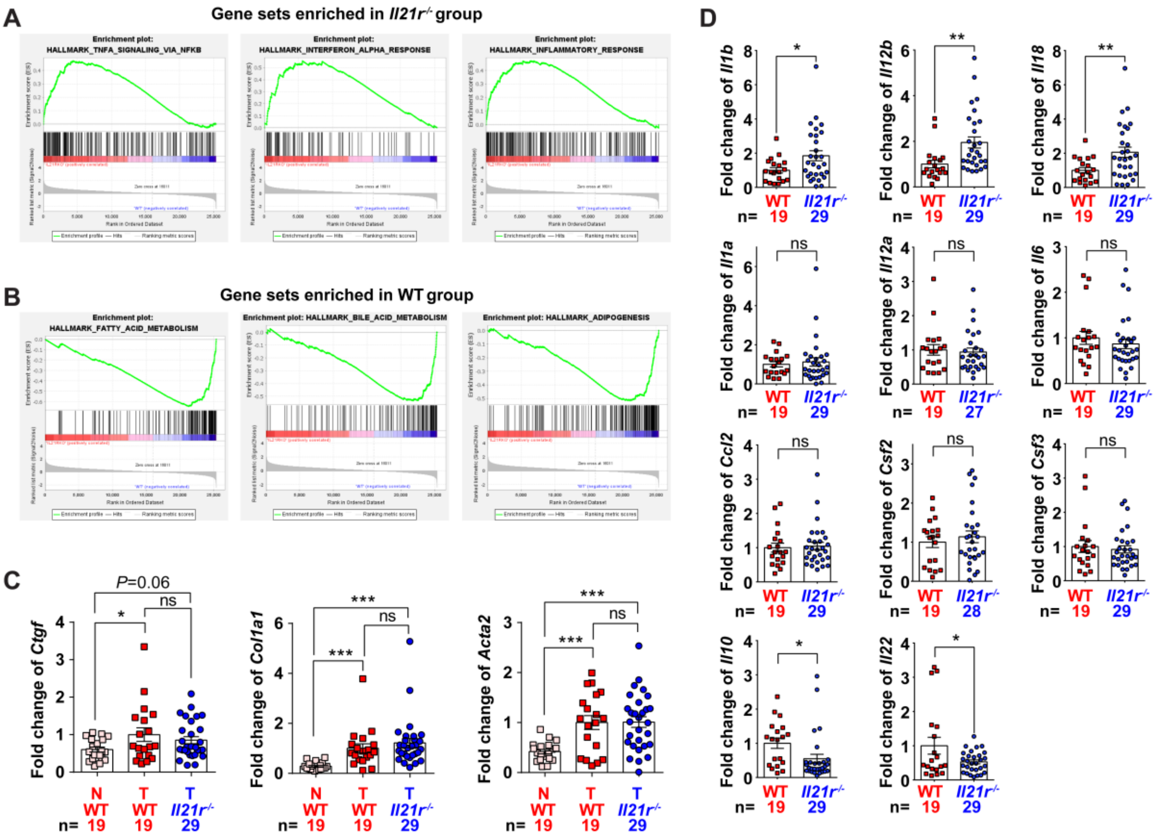

Figure S5. Gene sets enrichment analysis and the mRNA levels of fibrosis- or inflammation-related genes for the liver/tumor tissues from *Il21r*$^{-/-}$ mice or wild type controls. (**A, B**) Shown are the representative gene sets enriched in *Il21r*$^{-/-}$ mice (A) or wild type (WT) controls (B) according to gene set enrichment analysis. (**C**) The mRNA levels of fibrosis-related genes were detected in the paracancerous tissue (N), and cancer tissue (T) from *Il21r*$^{-/-}$ mice and WT controls. (**D**) The mRNA levels of pro- and anti-inflammatory cytokines and chemokines were detected in the cancer tissue from *Il21r*$^{-/-}$ mice and WT controls. The number of mice in C and D is shown. Student's *t* test was used to determine significance. * $P < 0.05$, ** $P < 0.01$, *** $P < 0.001$, ns, not significant difference.

Figure S6

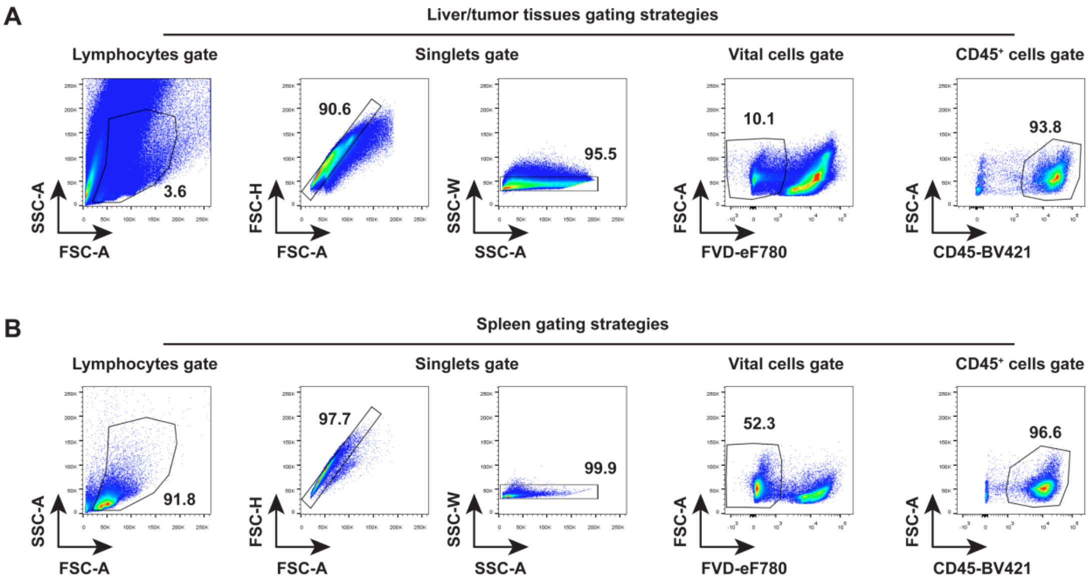

Figure S6. The gating strategies for liver lymphocytes and splenocytes. Liver/tumor cell suspensions (A) or single splenocyte (B) from tumor (HCC)-bearing mice were stained with CD45 and FVD-eF780, which was used to exclude dead cells. The sequential gating strategies for liver lymphocytes and splenocytes are as follows: lymphocyte gate, doublets exclusion, dead cell exclusion, and $CD45^+$ population gate.

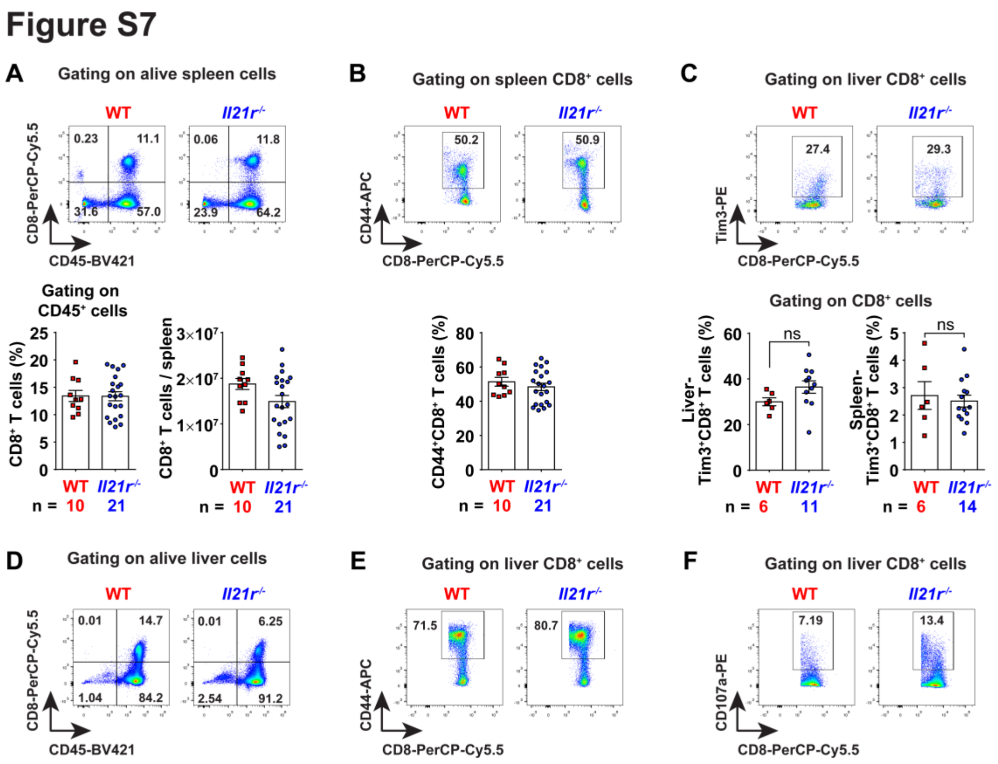

Figure S7. Liver/tumor effector and degranulating CD8$^+$ T cells are increased in *Il21r*$^{-/-}$ mice, whereas exhausted CD8$^+$ T cells are not changed. (**A**) Spleen CD8$^+$ T cells were not different between *Il21r*$^{-/-}$ mice and wild type (WT) controls in the STAM model. The representative flow cytometry images are shown in the upper panel. In the lower panel, the percentage of CD8$^+$ T cells and the absolute cell number of CD8$^+$ T cells per spleen were analyzed between *Il21r*$^{-/-}$ mice and WT controls. (**B**) The percentages of CD8$^+$ T cells positive for CD44 in the spleen were not different between *Il21r*$^{-/-}$ mice and WT controls in the STAM model. (**C**) Ablation of IL-21R did not change the percentage of exhausted CD8$^+$ T cells in the liver/tumor tissues or spleen. (**D-F**) Liver/tumor CD8$^+$ T cells were significantly decreased in *Il21r*$^{-/-}$ mice; however, liver/tumor effector and degranulating CD8$^+$ T cells were increased in *Il21r*$^{-/-}$ mice in the WD&High sugar solution&CCl_4 model. In D-F, *Il21r*$^{-/-}$ mice and WT controls were subjected to WD&High sugar solution&CCl_4 model. At the endpoint, the liver/tumor suspensions were stained with indicated markers, following flow cytometry.

Shown are the representative flow cytometry images. The gating scheme is indicated above each panel, and the number of mice in each group is shown in the panels accordingly. Student's *t* test was used to determine significance. ns, not significant difference.

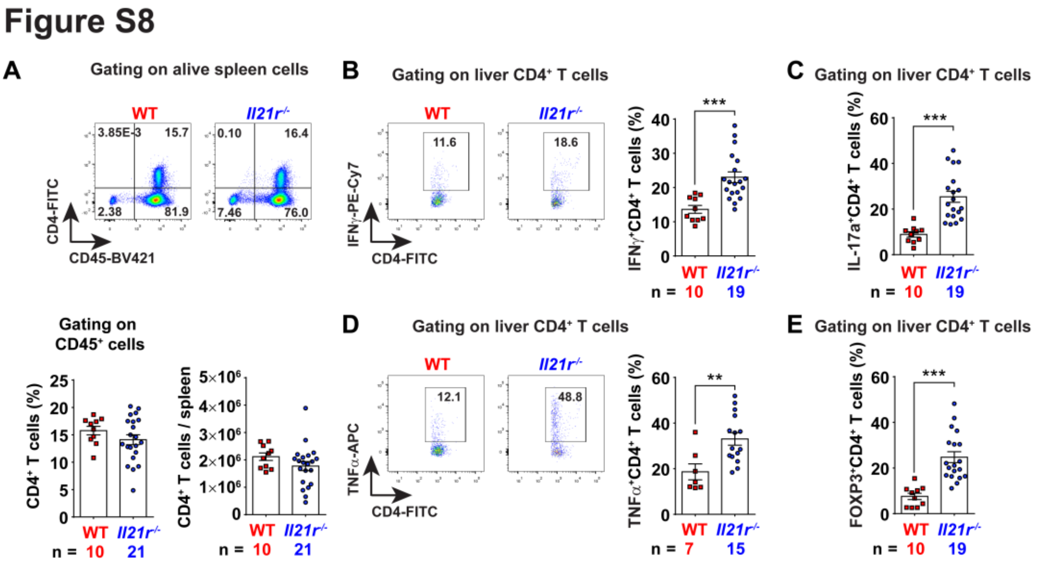

Figure S8. Th1, Th17 and Treg cells were significantly increased in the liver/tumor tissues of *Il21r*$^{-/-}$ mice in STAM model. (**A**) Ablation of IL-21R did not change the percentage and number of spleen CD4^{+} T cells. The percentage of CD4^{+} T cells and the absolute cell number of CD4^{+} T cells per spleen were analyzed between *Il21r*$^{-/-}$ mice and wild type (WT) controls. (**B-E**) Th1, Th17 and Treg cells were significantly increased in the liver/tumor tissues of *Il21r*$^{-/-}$ mice. The percentages of CD4^{+} T cells positive for IFNγ (B), IL-17a (C), TNFα (D) and FOXP3 (E) in liver/tumor tissues were analyzed between *Il21r*$^{-/-}$ mice and WT controls. Shown are the representative flow cytometry images and statistical results. The number of mice in each group is shown in the panels accordingly. Student's *t* test was used to determine significance. ** $P < 0.01$, *** $P < 0.001$.

Figure S9

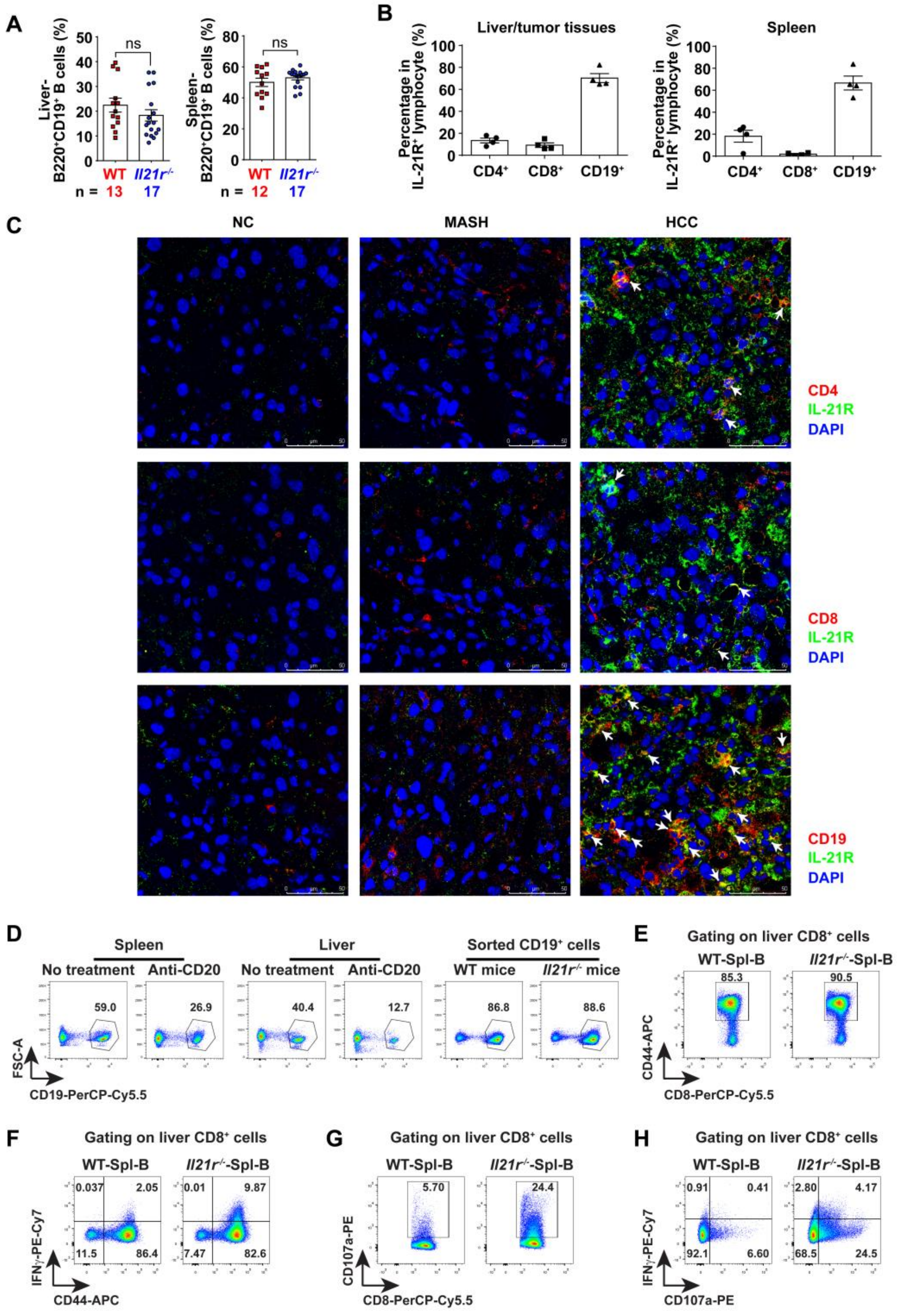

Figure S9. IL-21R is mainly expressed in the B cells of liver/tumor and spleen from HCC-bearing mice, and adoptive transfer of B cells from IL-21R-deficient mice into WT mice increases effector and degranulating CD8$^+$ T cells in recipient mice. (**A**) Ablation of IL-21R did not change the percentage of B220$^+$CD19$^+$ B cells in the liver/tumor tissues or spleen. (**B**) The IL-21R$^+$ population in CD45$^+$ lymphocytes were mainly CD19-positive. The

liver/tumor cell suspensions or splenocytes from HCC-bearing mice were stained with CD45, IL-21R, CD4, CD8, CD19 and FVD-eF780, following flow cytometry. The IL-21R$^+$ population in CD45$^+$ lymphocytes were subsequently analyzed for the percentages which were positive for CD4, CD8 and CD19, respectively. (**C**) IL-21R was increased in HCC and mainly co-localized with CD19, which is a marker of B cells. The liver tissues from mice at the indicated stages were stained for CD4, CD8 or CD19 (red) and IL-21R (green) by using immunofluorescent staining. Yellow signals indicate co-localization of IL-21R and the other three markers (white arrow). NC, normal chow. MASH, metabolic dysfunction-associated steatohepatitis. HCC, hepatocellular carcinoma. Scale bar = 50 μm. (**D**) The percentage of CD19$^+$ B cells for anti-CD20 treatment and the purity of sorted CD19$^+$ B cells were confirmed by flow cytometry. (**E-H**) Both effector and degranulating CD8$^+$ T cells were significantly increased in the liver/tumor tissues of mice transferred with B cells from *Il21r*$^{-/-}$ mice. The representative flow cytometry images are shown in E-H. The number of mice in each group is shown in panel A. Student's *t* test was used to determine significance. ns, not significant difference.

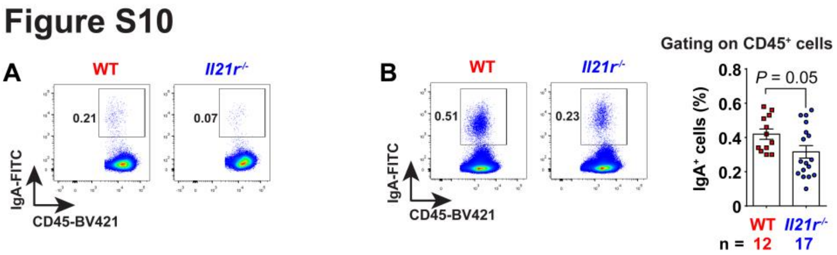

Figure S10. Ablation of IL-21R decreases the percentage of liver/tumor or spleen IgA$^+$ B cells. (**A**) Liver/tumor IgA$^+$ B cells were decreased in *Il21r*$^{-/-}$ mice in the WD&High sugar solution&CCl_4 model. (**B**) The percentage of spleen IgA$^+$ B cells was significantly decreased in *Il21r*$^{-/-}$ mice in the STAM model.

Figure S11

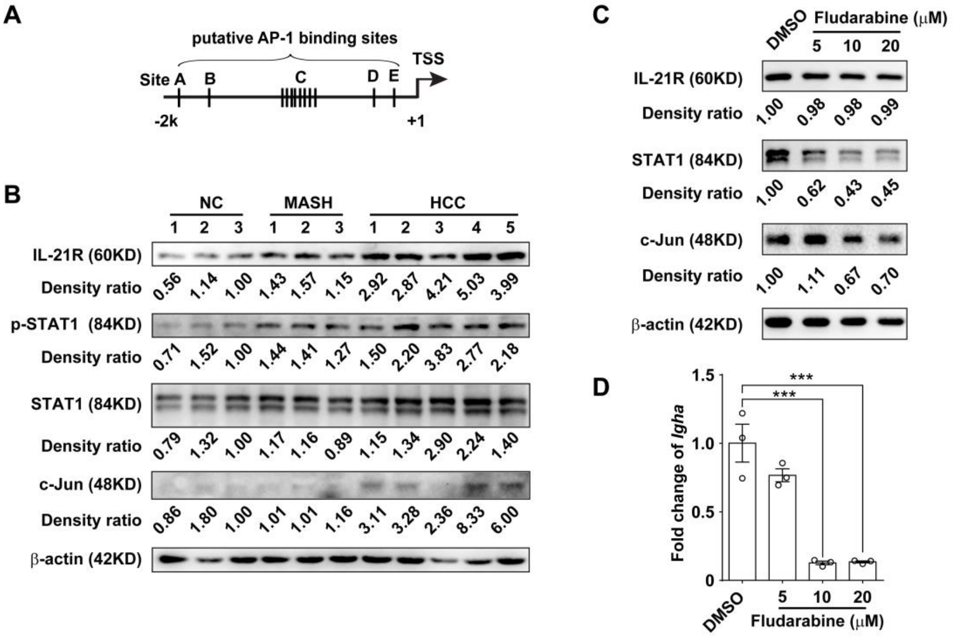

Figure S11. IL-21R-STAT1-c-Jun/c-Fos-IgA axis is activated during MASH-driven heptocacinogenesis. (**A**) Schematic diagram for AP-1 binding sites in the promoter region of *Igha*. The AP-1 binding sites between the 2-kb upstream of transcription start site (TSS) and TSS of *Igha* transcripts were predicted by Alibaba 2.1. Putative AP-1 binding sites are depicted as vertical lines. (**B**) IL-21R, phosphorylated STAT1, STAT1 and c-Jun were upregulated in the B cells from MASH-driven HCC. Purified $CD19^+$ B cells by using magnetic beads were obtained from the splenocytes from wild type mice at normal chow (NC), metabolic dysfunction-associated steatohepatitis (MASH) and hepatocellular carcinoma (HCC) stages, and subsequently subjected to western blotting. (**C**) Inhibition of STAT1 downregulated c-Jun without change the expression of IL-21R. (**D**) Inhibition of STAT1 decreased the mRNA levels of *Igha* in the B cells. In C and D, purified $CD19^+$ B cells by using magnetic beads were obtained from the splenocytes of mice at HCC stage, followed by treatment with STAT1 inhibitor, Fludarabine, at different dose for 24 hours. Thereafter, the

treated cells were subjected to western blotting (C) and qRT-PCR (D). β-actin, internal control. Student's *t* test was used to determine significance. *** $P < 0.001$.

Figure S12

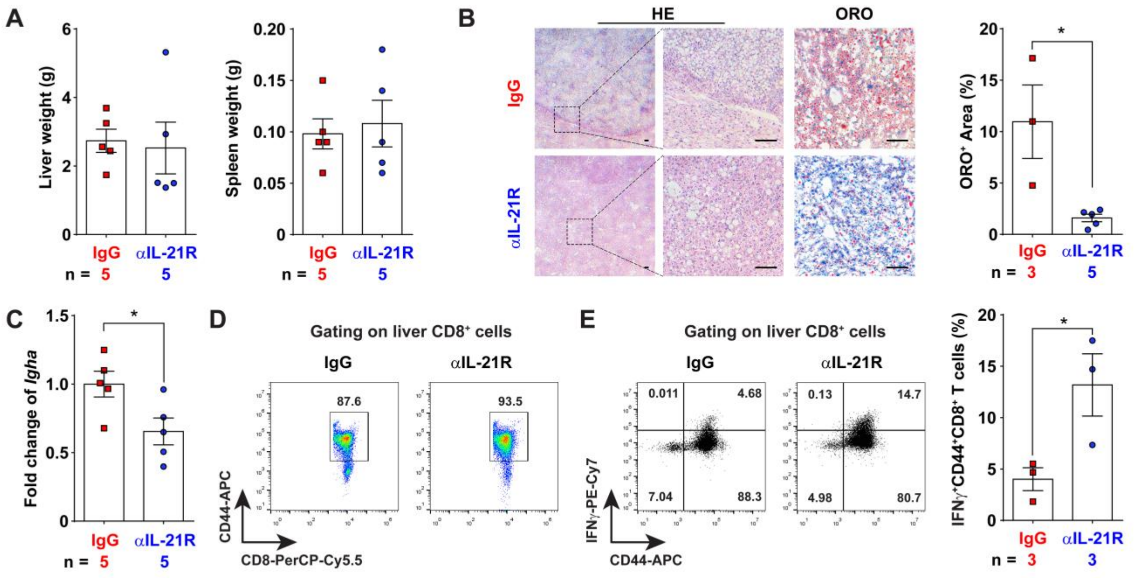

Figure S12. Blocking of IL-21R reduced lipid accumulation and enhanced cytotoxic CD8+ T lymphocyte activation in MASH-driven HCC. (**A**) Comparison of liver and spleen weight between the mice injected with IL-21R blocking antibody (αIL-21R) and its isotype control (IgG). (**B**) Blocking of IL-21R reduced lipid accumulation. Lipid droplets were quantified according to the image analysis of Oil Red O (ORO). (**C**) Blocking of IL-21R decreased the mRNA levels of *Igha* in the B cells. Purified CD19+ B cells by using magnetic beads were obtained from the splenocytes of mice injected with IL-21R blocking antibody or its isotype control, and subsequently subjected to qRT-PCR. (**D, E**) Effector CD8+ T cells were increased in the liver/tumor tissues of mice injected with IL-21R blocking antibody. The representative flow cytometry images of CD8+ T cells positive for CD44 (D), CD44 and IFNγ (E) in the liver/tumor tissues are shown. The number of mice in each group is shown in the panels accordingly. Student's *t* test was used to determine significance. * $P < 0.05$.
