# Supplementary material for: Interleukin-21 receptor signaling promotes metabolic dysfunction-associated steatohepatitis-driven hepatocellular carcinoma by inducing immunosuppressive IgA+ B cells
Source: Mol Cancer. 2024 May 8;23:95. doi: 10.1186/s12943-024-02001-2 (PMC11077880; doi:10.1186/s12943-024-02001-2)

Full uncropped Gels and Blots image

Note: The labels colored in red are the original labels, which can't be cut due to the integrity of blots.

Fig. 1F

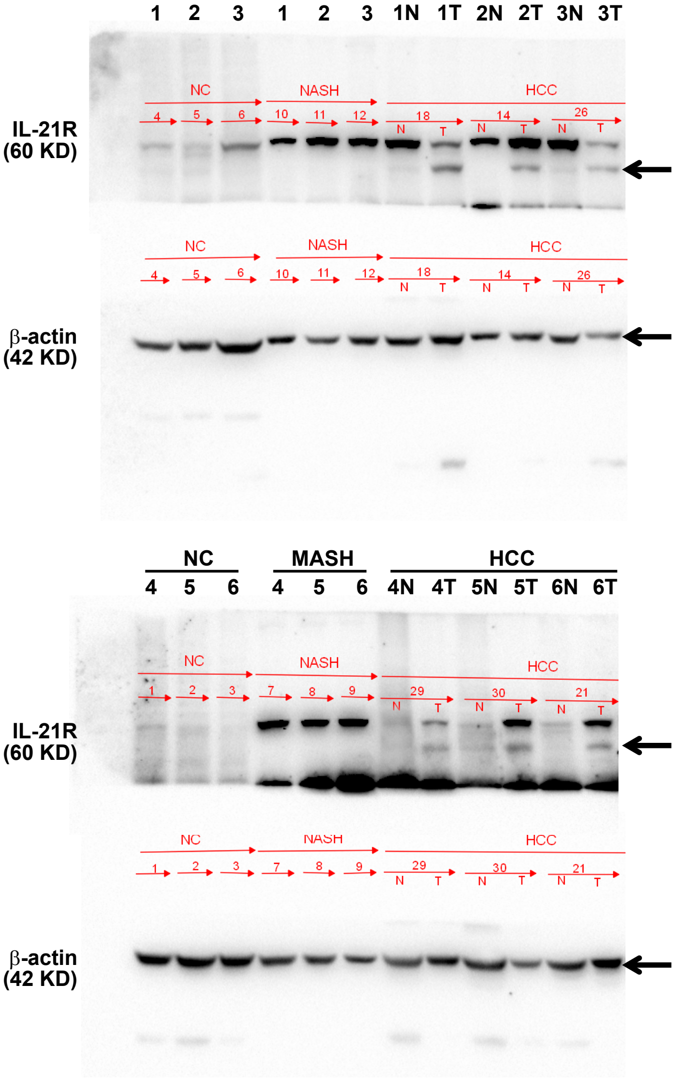

Fig. 6C

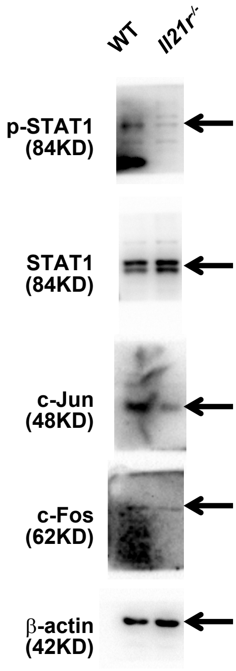

Fig. 6F

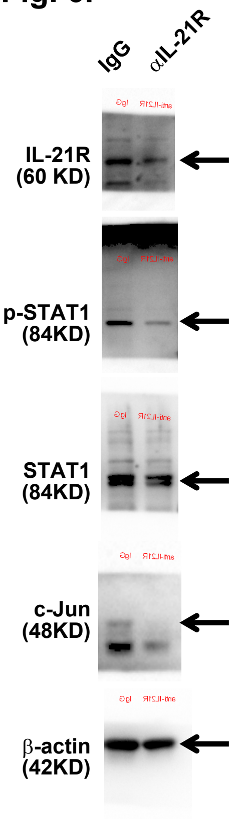

Fig. S3A

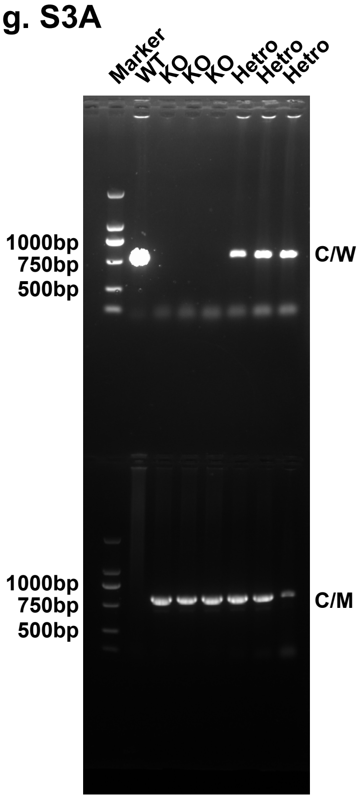

Fig. S11B

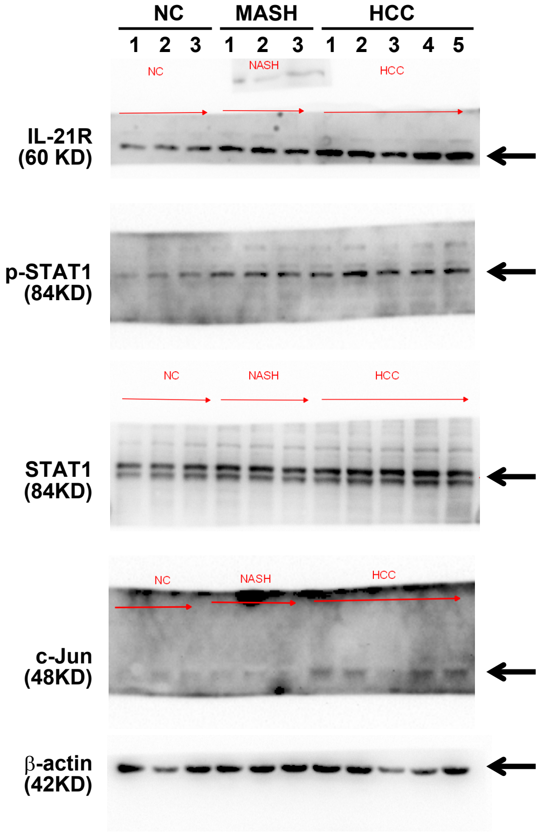

Fig. S11C

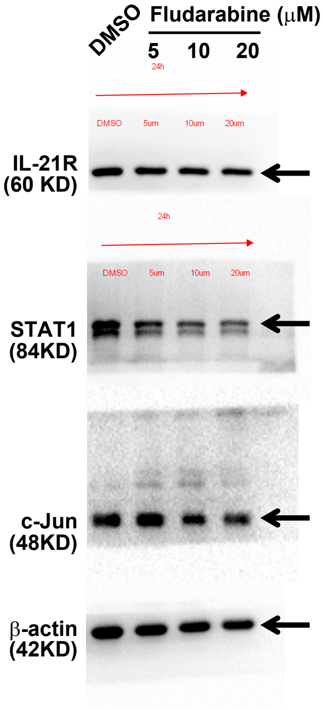

Supplement: Supplementary file 4 — Supplementary Material 4. [file 12943_2024_2001_MOESM4_ESM.pdf]
